# From Solvent Design to Biological Response: Structure–Property Relationships of Edible Natural Deep Eutectic Solvents for the Extraction of *Nigella sativa* Bioactives

**DOI:** 10.3390/molecules31162854

**Published:** 2026-08-15

**Authors:** Emina Mehmedović, Vesna B. Jovanović, Maja Krstić Ristivojević, Smilja Marković, Ivana Prodić, Husejin Keran, Katarina Smiljanić

**Affiliations:** 1 Faculty of Technology and Faculty of Natural Sciences and Mathematics, University of Tuzla, 75000 Tuzla, Bosnia and Herzegovina; emiina.mehmedovic@gmail.com; 2 CoE for Molecular Food Sciences, Department of Biochemistry, University of Belgrade—Faculty of Chemistry, Studentski Trg 12-16, 11158 Belgrade, Serbia; vjovanovic@chem.bg.ac.rs (V.B.J.); krstic_maja@chem.bg.ac.rs (M.K.R.); 3 Institute of Technical Sciences of SASA, Knez Mihailova 35/IV, 11000 Belgrade, Serbia; smilja.markovic@itn.sanu.ac.rs; 4 Institute of Virology, Vaccines and Sera “Torlak”, Vojvode Stepe 458, 11152 Belgrade, Serbia; iprodic@torlak.rs; 5 Faculty of Technology, University of Tuzla, 75000 Tuzla, Bosnia and Herzegovina; husejin.keran@untz.ba

**Keywords:** antioxidant activity, cytocompatibility, edible natural deep eutectic solvents (NADES), *Nigella sativa*, ready-to-use extracts, solvent design, specific energy consumption, structure–property relationships, total phenolic content, ultrasound-assisted extraction

## Abstract

Edible Natural Deep Eutectic Solvents (NADES) offer a route to ready-to-use extracts without solvent removal. This study examined how rational formulation design influences physicochemical properties, extraction performance, energy efficiency, thermal behavior, and cytocompatibility during bioactive recovery from *Nigella sativa* seeds. Twelve formulations spanning malic acid–polyol, citric acid–polyol, binary polyol, and ternary acid–polyol families were evaluated under standardized ultrasound-assisted conditions and compared with ethanolic ultrasound-assisted extraction, Soxhlet extraction, and cold-pressed oils. Selected NADES formulations outperformed the conventional systems. E1 (malic acid:glycerol:water) achieved the highest total phenolic content and lowest specific energy consumption (40.00 kJ mg^−1^ GAE), below the Soxhlet benchmark (55.17 kJ mg^−1^ GAE), whereas E9 (glycerol:xylitol:water) exhibited the greatest ABTS activity. Neat-NADES apparent pH and viscosity were inversely associated with total phenolic content, while viscosity and density were inversely associated with ABTS activity. ATR-FTIR showed stable, solvent-dominated fingerprints over 15 days, whereas DSC better differentiated neat NADES from their extracts. Most systems remained cytocompatible at 10,000× dilution, while acid–polyol formulations reduced viability at 500×; partial neutralization of N5/E5 restored viability. NADES performance was formulation- and application-dependent, requiring joint optimization of composition, acidity, viscosity, energy efficiency, and biologically compatible concentration.

## 1. Introduction

*Nigella sativa* L. (black cumin) is one of the most extensively investigated medicinal oilseed crops owing to its rich phytochemical composition and long-standing use in traditional medicine, functional foods, and nutraceutical applications [1]. Native to the Mediterranean region, Southwest Asia, and North Africa, its seeds contain a diverse array of biologically active constituents, including thymoquinone, phenolic acids, flavonoids, alkaloids, sterols, tocopherols, and unsaturated fatty acids, which collectively contribute to antioxidant, anti-inflammatory, antimicrobial [2], immunomodulatory, and metabolic health-promoting effects [3]. These biological activities have stimulated growing scientific and industrial interest in developing efficient strategies for recovering valuable bioactive compounds from *Nigella sativa* while preserving their chemical integrity and biological functionality [3,4,5,6,7,8].

Conventional solvent extraction, ultrasound-assisted extraction, and cold pressing remain the most frequently employed approaches, each offering distinct advantages depending on the target compounds. The physicochemical characteristics and microbiological stability of cold-pressed *Nigella sativa* oil are strongly influenced by storage temperature and conditions [9]. However, these techniques also present important limitations, including the use of volatile organic solvents, prolonged extraction times, high energy requirements, limited selectivity, or incomplete recovery of hydrophilic phytochemicals [10]. Consequently, the search for alternative extraction media that combine high extraction efficiency with improved environmental sustainability has become an important objective in the valorization of medicinal plants.

Natural Deep Eutectic Solvents (NADES), formed by naturally occurring metabolites linked through hydrogen-bond interactions, have emerged as promising green alternatives to conventional extraction solvents [11,12]. They combine biodegradability, low volatility, and tunable physicochemical properties with excellent solvating capacity for a broad range of plant bioactive compounds. Owing to their food-grade constituents, many NADES are considered edible solvents, making them particularly attractive for food, nutraceutical, cosmetic, and pharmaceutical applications, where the resulting NADES extracts may be used directly as ready-to-use formulations without complete solvent removal [11,13]. Increasing evidence indicates that NADES can enhance the extraction of phenolics and other phytochemicals while reducing reliance on hazardous organic solvents [14]. Importantly, their extraction performance depends strongly on solvent composition, since hydrogen-bond networks govern viscosity, polarity, acidity, and mass-transfer characteristics [15]. Consequently, increasing attention is being directed toward the rational design of solvent systems according to the physicochemical requirements of the target bioactive compounds.

Although the application of NADES for the extraction of plant bioactive compounds has expanded rapidly in recent years [16,17,18], studies specifically applying these solvents to *Nigella sativa* remain comparatively limited and have largely focused on extraction optimization or individual analytical endpoints [19]. Comparatively few studies have integrated NADES composition and physicochemical characteristics with extraction performance and the biological properties of the resulting extracts, while process-level sustainability has rarely been evaluated within the same experimental framework [14,20,21]. Furthermore, studies integrating solvent physicochemical characterization with spectroscopic and thermal analyses, energy-efficiency assessment, and cytocompatibility evaluation remain limited, particularly for edible NADES applied to *Nigella sativa* [19]. Addressing these relationships is essential for moving beyond empirical solvent selection toward the rational design of extraction systems tailored to the physicochemical requirements of target bioactive compounds and their intended applications.

Therefore, the aim of the present study was to investigate how the rational design of edible NADES influences physicochemical properties, extraction efficiency, process sustainability, and biological response during the recovery of bioactive compounds from *Nigella sativa* seeds. To this end, twelve edible NADES formulations based on citric and malic acids combined with naturally occurring polyols (glycerol, xylitol and sorbitol) were prepared and comprehensively characterized using physicochemical measurements, ATR-FTIR spectroscopy, and differential scanning calorimetry (DSC). The NADES were then employed in ultrasound-assisted extraction (UAE), and the resulting extracts were compared with an ethanolic UAE control, a conventional ethanolic Soxhlet extract, and cold-pressed oils in terms of total phenolic content and antioxidant activity. The oxidative quality indices and fatty acid composition of the cold-pressed oils were evaluated separately. In addition, extraction sustainability was evaluated using specific energy consumption (SEC), while the in vitro safety profile of the resulting extracts was assessed based on their cytocompatibility in HaCaT keratinocytes. Finally, correlation analysis was employed to establish structure–property relationships between solvent physicochemical characteristics and extraction performance, providing a rational framework for the design of edible NADES tailored to specific extraction objectives and intended applications.

## 2. Results

### 2.1. Physicochemical Characterization of Designed Binary and Ternary Edible NADES

To investigate how NADES solvent composition influences their physicochemical, extraction-related, thermal, and biological properties, twelve binary and ternary formulations were rationally designed using food-grade hydrogen-bond donors and acceptors. Four compositional families differing in the nature of the hydrogen-bonding components: malic acid-based systems (N1–N3), citric acid-based systems (N4–N6), binary polyol systems (N7–N9), and ternary acid-polyol systems (N10–N12) were prepared (Table 1). Both the polyols and organic acids used in this study contain oxygen-bearing functional groups that can, in principle, participate in hydrogen bonding as either donor or acceptor sites, although their effective roles depend on the composition and local molecular environment of each formulation [22]. Accordingly, the constituents are identified by their chemical identity rather than assigned rigid HBA or HBD designations in Table 1.

Malic and citric acids were selected as representative edible organic acids differing in carboxyl functionality, while glycerol, xylitol, and sorbitol were chosen as structurally related polyols with progressively increasing molecular weight, carbon-chain length, and number of hydroxyl groups. This experimental design enabled systematic comparison of solvent families with progressively increasing compositional complexity while maintaining exclusively edible constituents throughout the study. Rather than maximizing the number of formulations, the selected NADES were designed to capture representative compositional variations expected to influence solvent properties and, consequently, extraction behavior.

Although all formulations were prepared exclusively from six edible constituents, they differed substantially in apparent pH, density, and viscosity, whereas the refractive index varied only within a relatively narrow range (1.4366–1.4667). Binary systems containing organic acids exhibited the lowest apparent pH values (0.12–0.51), while binary polyol systems were considerably less acidic (pH 2.38–4.52). The ternary formulations displayed intermediate apparent pH values (1.76–1.86), reflecting the significantly reduced proportion of citric acid in their composition. Dynamic viscosity showed the greatest variability among the investigated properties, ranging from 15.07 to 94.18 mPa·s, indicating that relatively small compositional changes produced substantial differences in flow behavior.

While viscosity increased progressively across the glycerol-, xylitol-, and sorbitol-containing malic-acid formulations (N1–N3), the corresponding citric-acid systems (N4–N6) clustered within the narrower interval defined by the malic acid–xylitol and malic acid–sorbitol systems, indicating a less pronounced dependence on polyol identity. Each ternary formulation exhibited a lower dynamic viscosity than the corresponding binary system composed of the same two polyols, likely due to its higher water content (30 vs. 25 wt%), given the pronounced viscosity-reducing effect of water in NADES [23].

**Figure 1:**
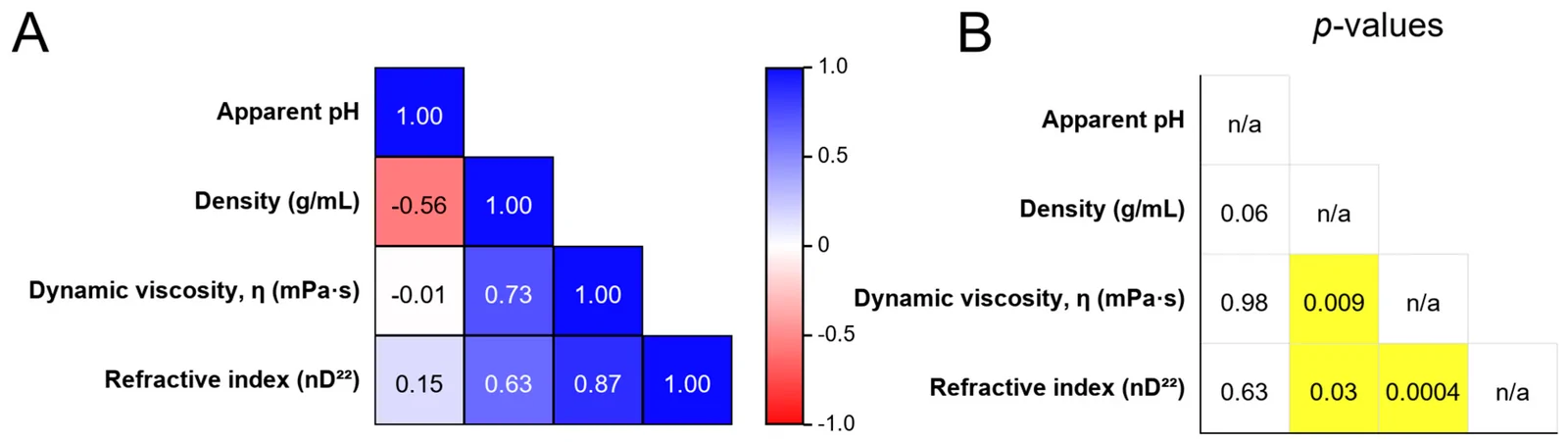
Spearman’s rank-correlation analysis of the physicochemical properties of the designed NADES formulations. (**A**) Correlation coefficient (ρ) matrix showing the direction and strength of associations among apparent pH, density, dynamic viscosity, and refractive index. (**B**) Corresponding *p*-values with statistically significant correlations (*p* < 0.05) are highlighted in yellow.

Spearman’s rank-correlation analysis (Figure 1A,B) revealed significant positive associations of dynamic viscosity with density (ρ = 0.73, *p* = 0.009) and refractive index (ρ = 0.87, *p* < 0.001), while density was also positively correlated with refractive index (ρ = 0.63, *p* = 0.03). Apparent pH showed a moderate inverse association with density (ρ = −0.56, *p* = 0.06), although this relationship did not reach statistical significance.

The molar ratio of water to total non-water components (H_2_O:NWC) was used as a molar hydration descriptor. Its association with dynamic viscosity was weak and non-significant when all twelve formulations were considered (ρ = 0.33, *p* = 0.30; Figure S1A). However, analysis restricted to the binary formulations with a constant water content of 25 wt% (N1–N9) revealed a strong positive association (ρ = 0.78, *p* = 0.017; Figure S1B), whereas inclusion of the ternary formulations containing 30 wt% water attenuated this relationship.

Collectively, these physicochemical data establish the initial solvent landscape against which the subsequent extraction performance, specific energy consumption, thermal behavior, antioxidant activity, and biological responses of the NADES formulations and their extracts can be interpreted.

### 2.2. ATR-FTIR Characterization of Designed NADES and Their Corresponding Nigella sativa Extracts

ATR-FTIR spectroscopy was first used to characterize the designed NADES formulations and assess spectral changes consistent with the establishment of intermolecular hydrogen-bonding networks. Subsequently, the technique was used to evaluate whether the extraction of *Nigella sativa* constituents and storage at room temperature or under refrigerated conditions altered the molecular vibrational fingerprints of the resulting NADES-based extracts. Conventional ethanolic extracts obtained by UAE and Soxhlet extraction were included as reference systems for comparison.

**Figure 2:**
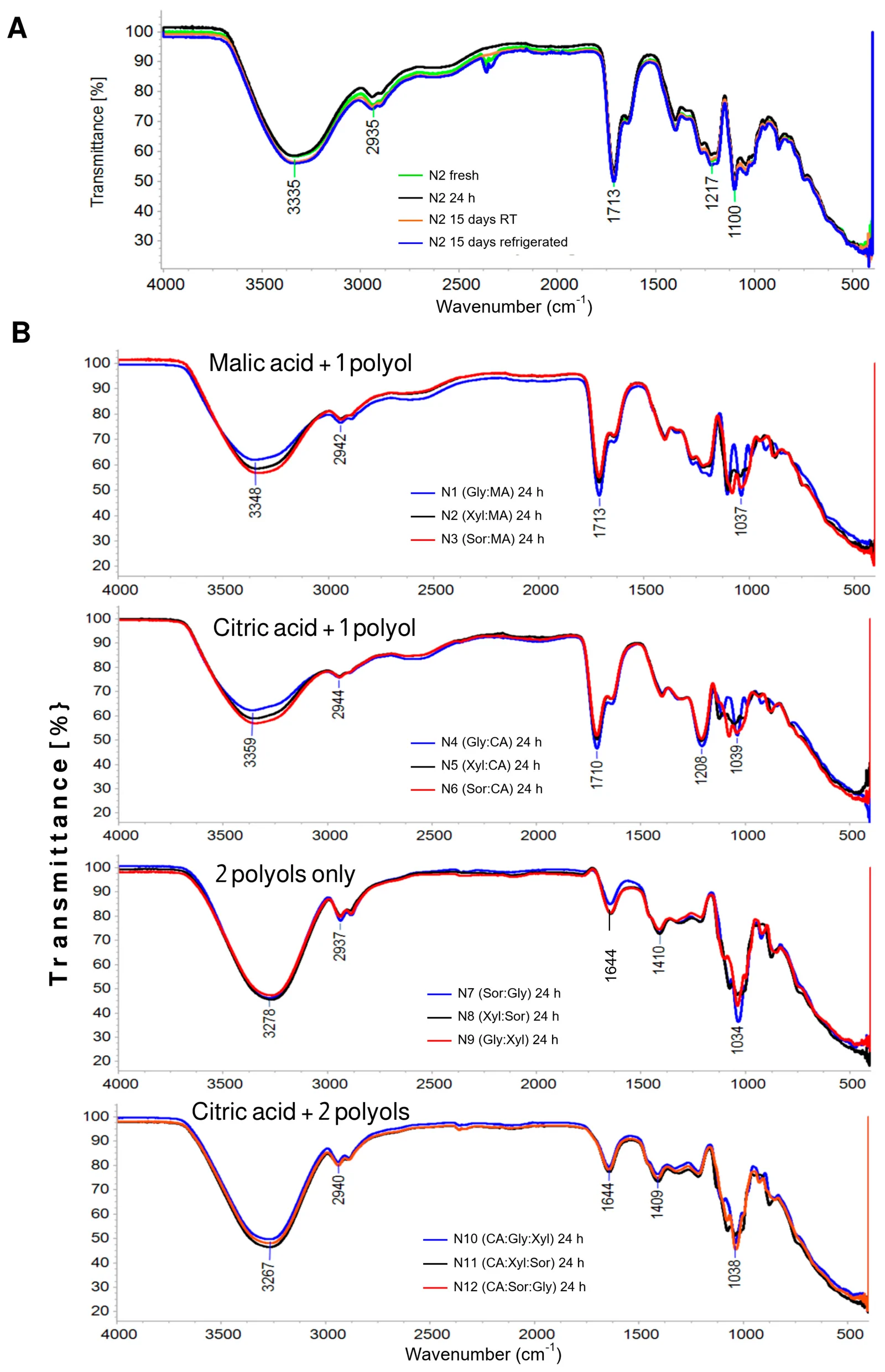
ATR-FTIR transmittance spectra of the designed NADES formulations. (**A**) Representative spectra of neat N2 recorded immediately after preparation, at 24 h, and at 15 days under either room temperature or refrigerated conditions. N2 was selected because it exhibited the most noticeable, although still limited, spectral variation among the investigated formulations. (**B**) Comparative overlays of the four NADES families after 24 h: malic acid–polyol formulations (N1–N3), citric acid–polyol formulations (N4–N6), binary polyol formulations (N7–N9), and ternary citric acid–polyol formulations (N10–N12). Abbreviations: Gly, glycerol; Xyl, xylitol; Sor, sorbitol; MA, malic acid; CA, citric acid.

The ATR-FTIR spectra of the pure starting components (Figure S2) served as reference profiles for assigning the principal absorption bands observed in the corresponding NADES formulations. For malic and citric acids, the characteristic absorptions included broad O–H stretching bands within the 2500–3300 cm^−1^ region, strong C=O stretching bands at approximately 1700–1725 cm^−1^, C–O stretching bands within the 1210–1320 cm^−1^ region, and an O–H out-of-plane bending band near 920 cm^−1^. The polyols were characterized by broad O–H stretching bands within the 3200–3400 cm^−1^ region, C–H stretching bands at approximately 2850–2950 cm^−1^, strong C–O stretching bands within the 1030–1150 cm^−1^ region, and additional O–H deformation bands within the 1330–1420 and 600–700 cm^−1^ regions. A representative example of the spectral stability of a neat NADES during storage is shown for N2 (Figure 2A), selected as the formulation exhibiting the most noticeable, yet still limited, spectral variation among the investigated systems. The complete set of spectra recorded immediately after preparation, after 24 h, after 15 days at room temperature, and after 15 days under refrigeration is presented in Figure S3, while Figure 2B provides a comparative overview of the four designed NADES families after 24 h.

The ATR-FTIR spectra of the neat NADES formulations displayed the characteristic broad O–H stretching band in the 3260–3360 cm^−1^ region together with intense absorptions within the 1034–1100 cm^−1^ fingerprint region. Relative to the pure components, broadening and shifts in the O–H stretching bands, together with changes within the 1500–600 cm^−1^ fingerprint region, were consistent with altered intermolecular hydrogen-bonding environments following NADES formation. The carbonyl absorptions of the binary acid–polyol formulations N1–N6 remained clearly resolved near 1710–1713 cm^−1^ [24], whereas the lower citric acid proportion in N10–N12 weakened its carbonyl contribution and prevented the appearance of a distinct absorption minimum. The feature near 1644 cm^−1^, which was also observed in the acid-free formulations N7–N9 and in deionized water (Figure S2), was interpreted primarily to the H–O–H bending vibration of water reported near 1640–1644 cm^−1^ [25,26], although minor overlapping contributions from other matrix vibrations cannot be completely excluded.

The binary polyol systems (N7–N9) were dominated primarily by O–H- and C–O-related absorptions. The O–H stretching maxima clustered according to the designed NADES families. Malic acid-based systems exhibited maxima between 3328 and 3335 cm^−1^, citric acid-based binary systems between 3346 and 3358 cm^−1^, binary polyol systems between 3266 and 3277 cm^−1^, and the ternary formulations between 3274 and 3277 cm^−1^. Across all storage conditions, the principal band positions remained essentially unchanged, while only minor variations in relative band intensity were observed, indicating high spectral consistency of the prepared NADES formulations (Figure 2A and Figure S3).

Following characterization of the neat solvents, ATR-FTIR analysis was extended to the corresponding ultrasound-assisted *Nigella sativa* extracts. Compared with their parent NADES, the extracts exhibited a high degree of spectral overlap, with no new absorption bands or substantial shifts in the characteristic vibrational regions (Figure S4). The spectra remained dominated by the vibrational features of the NADES matrix, while only modest differences in relative band intensities were observed following extraction and during storage. In parallel, all NADES extracts exhibited higher apparent pH values than the corresponding neat solvents (Table S1), indicating a shift in the operationally measured acidity following incorporation of seed-derived constituents.

The conventional ethanolic extracts obtained by UAE (E13) and Soxhlet extraction (E14) displayed broadly similar ATR-FTIR profiles, characterized by hydroxyl stretching bands and absorptions within the fingerprint region typical of plant-derived constituents. The available spectra likewise exhibited extensive overlap, with only minor differences in relative band intensities between the ultrasound-assisted and Soxhlet extracts after storage (Figure S4).

Overall, the ATR-FTIR results supported the establishment of composition-dependent intermolecular interactions within the designed NADES and showed that neither extraction nor storage produced substantial changes in their molecular vibrational fingerprints. The extensive spectral overlap between neat NADES and their corresponding extracts indicates that the dominant spectral contribution originated from the solvent matrix, making discrimination of the extracted constituents by ATR-FTIR alone challenging. The individual ATR-FTIR spectra underlying these comparisons are openly available in the associated Zenodo dataset [27]. This observation provided the rationale for complementary thermal characterization by DSC, presented in the following section.

### 2.3. Comparative DSC Profiling of Neat NADES and Their Corresponding Extracts

**Figure 3:**
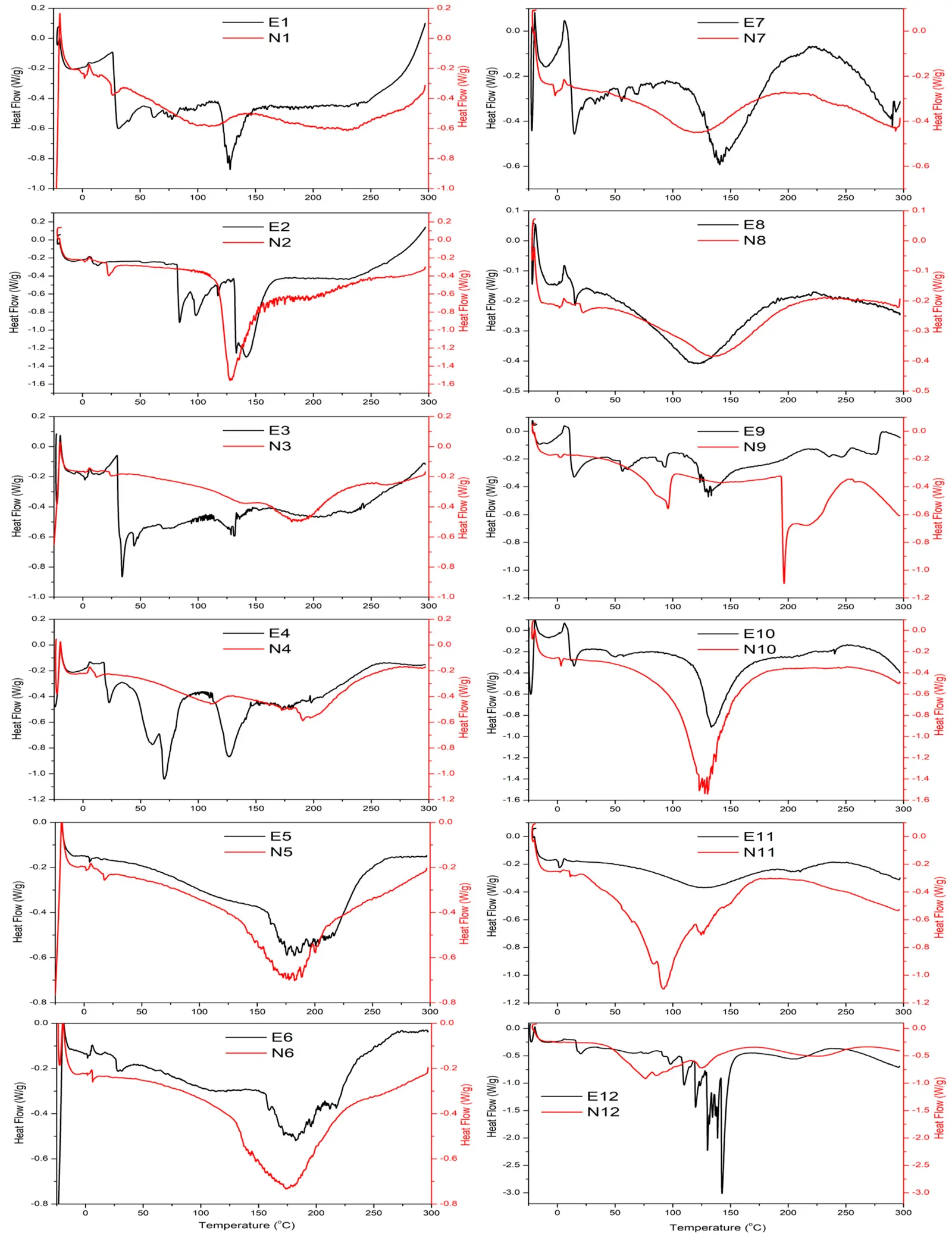
Paired DSC thermograms of the neat NADES formulations (N1–N12; red curves, right heat-flow axis) and their corresponding *Nigella sativa* extracts (E1–E12; black curves, left heat-flow axis) after 15 days of refrigerated storage. Thermograms were recorded over the temperature range from −20 to 300 °C using the exothermic-up convention. Summary thermal-profile descriptors are presented in Table 2, while complete integration intervals, peak temperatures, and integrated heat values are provided in Table S2.

DSC was used to compare the thermal profiles of the neat NADES formulations and their corresponding *Nigella sativa* extracts after 15 days of refrigerated storage (Figure 3). The thermograms revealed composition-dependent differences in the number and distribution of annotated thermal events, while the extracts exhibited distinct changes in their overall thermal profiles relative to the corresponding neat formulations. Table 2 summarizes these differences using the number of annotated events and the temperature of the dominant event, operationally defined as the event with the largest absolute integrated heat within each thermogram. Complete integration intervals, peak temperatures, and integrated heat values are provided in Table S2.

The thermal response following extraction was not uniform across the investigated formulations. The dominant-peak temperature was nearly unchanged for N5/E5 and N10/E10, with Δ*T*_peak_ values of −0.1 and +3.4 °C, respectively, although the number of annotated events changed in both pairs. Conversely, N3/E3, N6/E6, N7/E7, N10/E10, and N11/E11 exhibited a greater number of events in the extract thermograms than in the corresponding neat NADES. This increased profile complexity is consistent with the presence of additional thermally distinguishable domains or processes within the resulting multicomponent extract systems. The largest negative differences in dominant-peak temperature were observed for N4/E4 and N9/E9 (Δ*T*_peak_ = −120.3 and −65.6 °C, respectively), whereas N11/E11 and N12/E12 displayed the largest positive differences (+34.5 and +65.9 °C, respectively).

These contrasting responses demonstrate that the thermal changes associated with extraction depended strongly on the composition of the parent NADES. Even within the same formulation family, the direction and magnitude of the difference in dominant-peak temperature and the change in the number of events varied with polyol identity. Previous calorimetric and spectroscopic investigations of citric acid–xylitol mixtures have similarly demonstrated that their phase and thermal behavior depend strongly on mixture composition and the resulting intermolecular associations [28]. The ternary systems were likewise not uniform: the dominant-peak temperature was almost unchanged in N10/E10 but occurred at considerably higher temperatures in the E11 and E12 extracts than in their corresponding neat formulations.

Because the ternary formulations also contained a higher water content than the binary systems (30 vs. 25 wt%), their thermal profiles reflect the combined influence of organic composition, water content, and incorporated seed constituents. Water is known to modify the hydrogen-bonding environment and physicochemical behavior of NADES, with the magnitude and nature of these effects depending on both water content and solvent composition [23]. Studies of a hydrated malic acid–choline chloride DES have further shown that water incorporation does not necessarily result in the complete loss of the underlying solvent organization, although the structural response to hydration remains system-dependent [29].

The calculated Δ*T*_peak_ values should therefore be regarded as comparative descriptors and do not imply that the dominant events in a neat NADES and its corresponding extract represent an identical physical transition. Likewise, the individual thermal events were not assigned to specific physical processes without additional supporting analyses. Overall, DSC differentiated the neat NADES from their corresponding extracts more clearly than ATR-FTIR spectroscopy, revealing extraction-associated thermal reorganization that was not readily apparent from the largely overlapping vibrational spectra.

### 2.4. Physicochemical Quality, Oxidative Status, and Fatty Acid Composition of Cold-Pressed Nigella sativa Oils

Cold-pressed oils were included as an additional and practically relevant form of *Nigella sativa* seed-derived preparation, obtained by mechanical recovery rather than solvent extraction and widely used for oral and topical applications. They therefore served both as reference lipid matrices and as comparators representing another route by which bioactive seed constituents are transferred into a ready-to-use product. In this context, cold-pressed oils were considered a more application-relevant comparator than a water-only extract. A water-only extract was not selected because the stability of its extracted constituents over time would be uncertain, particularly given the rapid degradation of thymoquinone in aqueous media [30]. Five oils, comprising one in-house preparation and four commercial products, were evaluated. Previous work has demonstrated that the physicochemical characteristics and stability of cold-pressed *Nigella sativa* oil can be strongly influenced by storage temperature and conditions [9].

All five oils were assessed for flow properties, antioxidant response using the oil-compatible DPPH and FRAP assays, and conventional oxidative quality indices. The in-house oil and Oil 3 were selected for additional analysis because they contained the greatest visible abundance of suspended seed particles among the investigated samples. This seed-derived particulate fraction was selected for further analysis in a parallel proteomic and immunoinformatic study addressing potential allergen candidates in *Nigella sativa* seeds and oil [31]. The two selected oils were additionally characterized for moisture and water content, refractive index, and fatty acid composition by GC-FID before and after centrifugation.

**Figure 4:**
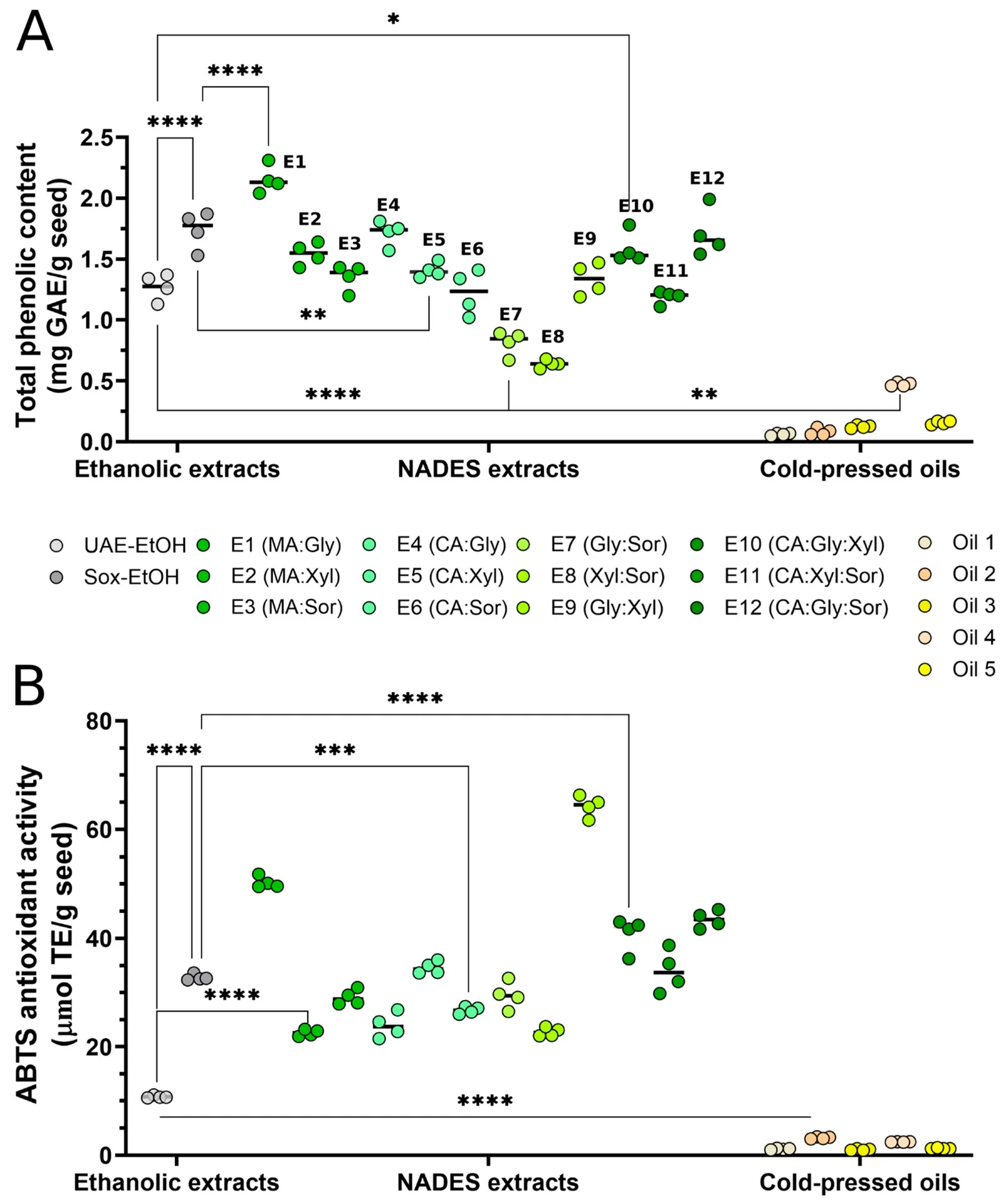
Comparative evaluation of *Nigella sativa* extracts and cold-pressed oils. (**A**) Total phenolic content (TPC), expressed as milligrams of gallic acid equivalents per gram of seed (mg GAE/g seed). (**B**) ABTS antioxidant activity, expressed as micromoles of Trolox equivalents per gram of seed (µmol TE/g seed). Data points represent individual replicate measurements for the ultrasound-assisted ethanolic extract (UAE–EtOH), Soxhlet ethanolic extract (Sox–EtOH), NADES-based extracts (E1–E12), and cold-pressed oils (Oil 1–5); thick horizontal bars indicate group medians. Statistical differences were assessed by one-way ANOVA followed by Šídák’s multiple-comparisons test. To avoid excessive overlap of significance brackets, the displayed comparisons were selected as boundary comparisons, identifying the samples closest to the ethanolic references among groups showing the same direction of statistically significant difference. Complete Šídák-adjusted comparisons against Sox–EtOH are provided in Table S6, while the full pairwise analysis is available in the associated GraphPad Prism project (*, *p* < 0.05; **, *p* < 0.01; ***, *p* < 0.001; and ****, *p* < 0.0001). MA, malic acid; CA, citric acid; Gly, glycerol; Xyl, xylitol; Sor, sorbitol.

Overall, the oils exhibited relatively similar flow properties but considerable variability in DPPH radical-scavenging activity, ferric reducing antioxidant power, and oxidative quality indices, while the two selected oils showed the characteristic fatty acid profile dominated by linoleic, oleic, and palmitic acids [32]. Detailed analytical procedures are provided in Supplementary Sections S1.1–S1.3, and the complete results are presented in Supplementary Sections S2.4–S2.5 and Tables S3–S5. For direct comparison across all investigated extraction systems, TPC and ABTS were determined for the NADES extracts, ethanolic extracts, and cold-pressed oils using a common 96-well microplate format, as presented in Section 2.5 and Figure 4.

### 2.5. Comparative Phenolic Recovery and Antioxidant Activity of NADES Extracts, Ethanolic Extracts, and Cold-Pressed Oils

The performance of the extraction approaches investigated was evaluated by determining the total phenolic content (TPC) and antioxidant activity. These complementary parameters were used to assess how solvent composition influenced the recovery of antioxidant constituents from *Nigella sativa* seeds and to compare the designed edible NADES with conventional ethanolic extraction and cold pressing.

Marked solvent-dependent differences were observed in both phenolic recovery and antioxidant activity (Figure 4). To preserve graphical clarity, only selected significance brackets were displayed, identifying the samples closest to the ethanolic reference values on either side (higher/lower) of a statistically significant difference. Complete numerical results, including mean ± SD values for TPC and ABTS activity and adjusted *p*-values for comparisons with Soxhlet extraction, are provided in Table S6.

As shown in Figure 4A, E1 exhibited the highest TPC among all investigated extracts and was significantly higher than both the ultrasound-assisted and Soxhlet ethanolic extracts. In contrast, E8 exhibited the lowest TPC among the NADES extracts and was significantly lower than both reference ethanolic extracts. However, to preserve graphical clarity, the significance bracket is displayed only for E7, the closest NADES-based extract that still differed significantly from both the UAE and Soxhlet ethanolic reference extracts (Figure 4A, Table S6). The remaining NADES formulations exhibited intermediate and composition-dependent phenolic recoveries, demonstrating that NADES performance could not be generalized across the entire group. The cold-pressed oils exhibited substantially lower TPC than all solvent-based extracts, reflecting the limited transfer of phenolic constituents into the oil fraction.

In the ABTS assay (Figure 4B), E9 showed the highest antioxidant activity, followed by E1, while E1, E9, E10, and E12 significantly outperformed the Soxhlet extract; the remaining NADES extracts showed comparable or lower activity. Ultrasound-assisted ethanolic extraction showed significantly lower TPC and ABTS values than Soxhlet extraction. Similar to TPC results, the cold-pressed oils exhibited the lowest antioxidant activity among the sample types investigated.

Taken together, comparison of the three extraction strategies clearly demonstrated that no conventional reference system consistently outperformed the best-designed edible NADES formulations. E1 achieved the highest TPC, E9 exhibited the greatest ABTS activity, whereas the cold-pressed oils consistently showed the lowest values in both assays. The conventional ethanolic extracts occupied an intermediate position, emphasizing that extraction performance depended on the specific formulation and extraction strategy rather than on the solvent category alone.

**Figure 5:**
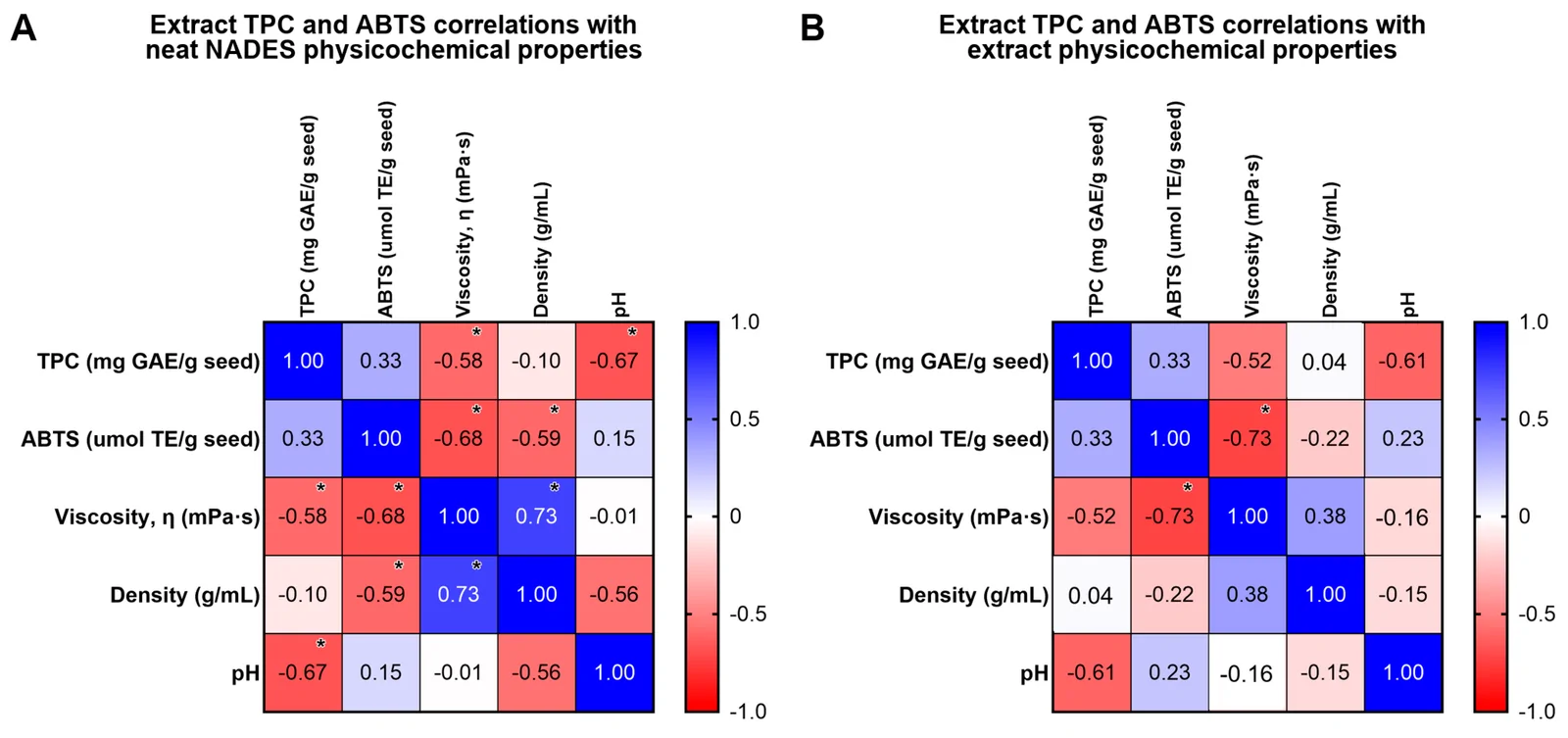
Spearman correlation matrices relating the total phenolic content (TPC) and ABTS antioxidant activity of NADES-based *Nigella sativa* extracts to physicochemical properties measured in the neat NADES or in the corresponding extracts (*n* = 12). (**A**) TPC and ABTS values of the extracts correlated with the dynamic viscosity, density, and apparent pH of the neat NADES formulations. (**B**) TPC and ABTS values correlated with the dynamic viscosity, density, and apparent pH of the corresponding NADES-based extracts. Spearman’s rank-correlation coefficients (ρ) are displayed within the cells and range from −1 to 1; positive correlations are shown in blue and negative correlations in red. Statistically significant correlations are marked with an asterisk, * (*p* < 0.05). TPC, total phenolic content, expressed as mg gallic acid equivalents per gram of seed (mg GAE/g seed); ABTS, antioxidant activity expressed as µmol Trolox equivalents per gram of seed (µmol TE/g seed).

Since the designed formulations differed markedly in pH, viscosity, density, and hydrogen-bonding characteristics (Section 2.1), a correlation analysis was performed to identify which solvent properties were most closely associated with phenolic recovery and antioxidant activity. Furthermore, because extraction altered certain physicochemical properties of the NADES systems, correlations were evaluated using both the original (neat) solvent properties and the corresponding extract properties to determine which set of measurements was more closely associated with extraction performance (Figure 5).

Among the investigated parameters, apparent pH and viscosity showed the closest associations with extraction performance. In the neat-NADES dataset (Figure 5A), TPC was inversely correlated with apparent pH (ρ = −0.67) and viscosity (ρ = −0.58), whereas ABTS activity showed inverse correlations with viscosity (ρ = −0.68) and density (ρ = −0.59). When the corresponding extract properties were considered (Figure 5B), several associations were attenuated, including TPC–viscosity (ρ = −0.52), TPC–apparent pH (ρ = −0.61), and particularly ABTS–density (ρ = −0.22). In contrast, the inverse association between ABTS activity and viscosity remained comparatively strong and became slightly more pronounced (ρ = −0.73).

The correlation matrix based on neat-NADES properties generally revealed stronger and more consistent associations with TPC and ABTS activity than the matrix based on the corresponding extract properties. This finding suggests that the physicochemical characteristics established during solvent design may be more informative for anticipating extraction performance than measurements obtained after extraction, although the observed associations should not be interpreted as evidence of causality.

The positive correlation between TPC and ABTS activity was weak-to-moderate in NADES-based extracts (ρ = 0.33), indicating only a modest tendency for extracts with higher phenolic content to exhibit greater antioxidant activity. This limited correspondence further indicates that antioxidant activity was not governed solely by total phenolic recovery but also likely depended on the composition and relative activities of the extracted constituents. Collectively, these findings identify viscosity and acidity as relevant formulation-level characteristics associated with the recovery of phenolic constituents and ABTS-active compounds.

#### Energy Efficiency of Standardized Ultrasound-Assisted Extraction

To complement the evaluation of phenolic recovery and antioxidant activity, the energy efficiency of the extraction protocols was assessed using specific energy consumption (SEC), expressed as the estimated energy input, calculated from the operating power and extraction time, per milligram of recovered gallic acid equivalents (kJ mg^−1^ GAE). Because all ultrasound-assisted extractions were conducted under the same standardized operating conditions, differences in SEC among these systems primarily reflected differences in phenolic recovery associated with solvent composition.

The conventional Soxhlet extraction had an estimated energy input of 2880 kJ during the 4 h extraction period, corresponding to an SEC of 55.17 kJ mg^−1^ GAE, whereas the standardized ultrasound protocol had an estimated input of only 345.6 kJ per extraction cycle, representing an approximately 8.3-fold lower energy input. Nevertheless, SEC varied markedly among solvent systems. The ultrasound-assisted ethanolic extraction exhibited an SEC of 67.50 kJ mg^−1^ GAE, exceeding that of the Soxhlet benchmark despite its substantially lower energy input.

**Figure 6:**
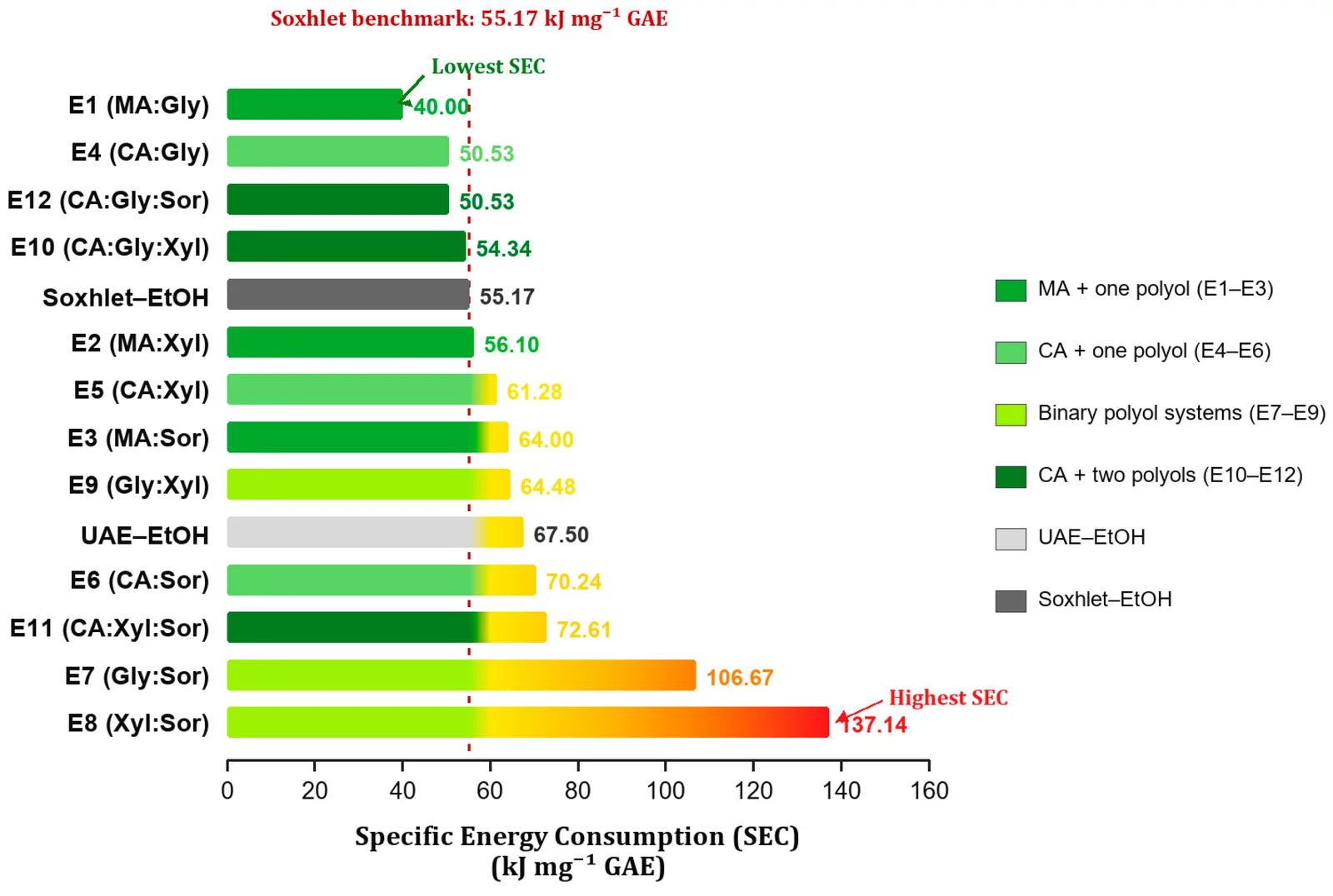
Specific energy consumption (SEC) of the investigated extraction protocols, expressed as the estimated energy input, calculated from the operating power and extraction time, per milligram of recovered gallic acid equivalents (kJ mg^−1^ GAE). Ultrasound-assisted extractions were performed under identical operating conditions (37 kHz, 192 W, 30 min, solid-to-solvent ratio 1:10, temperature maintained below 40 °C), allowing direct comparison of solvent performance independent of extraction parameters. Bars are arranged in ascending order of SEC and colored consistently with Figure 4 according to NADES compositional family and ethanolic extraction controls; MA, malic acid; CA, citric acid; Gly, glycerol; Xyl, xylitol; Sor, sorbitol; UAE–EtOH, ultrasound-assisted ethanolic extraction; Soxhlet–EtOH, Soxhlet ethanolic extraction. The dashed vertical line denotes the SEC of the conventional Soxhlet extraction (55.17 kJ mg^−1^ GAE), which served as the reference benchmark. For protocols exceeding this benchmark, the yellow-to-red bar extensions indicate a progressively greater excess in SEC. Lower SEC values indicate greater extraction energy efficiency.

Among the investigated NADES-based extraction systems, SEC values ranged from 40.00 to 137.14 kJ mg^−1^ GAE (Figure 6). E1 exhibited the lowest SEC (40.00 kJ mg^−1^ GAE), followed by E4 and E12 (50.53 kJ mg^−1^ GAE) and E10 (54.34 kJ mg^−1^ GAE), all of which outperformed the Soxhlet benchmark. Within both acid–polyol families (E1–E3 and E4–E6), SEC increased consistently in the order glycerol < xylitol < sorbitol. A related composition-dependent pattern was observed among the binary polyol systems: the glycerol–xylitol formulation E9 exhibited a lower SEC than glycerol–sorbitol E7, whereas the xylitol–sorbitol formulation E8 showed the highest SEC among all investigated systems. Notably, introducing citric acid at a molar proportion of 0.1 into each binary polyol formulation consistently reduced SEC, from 106.67 to 50.53 kJ mg^−1^ GAE for the glycerol–sorbitol pair (E7/E12), from 137.14 to 72.61 kJ mg^−1^ GAE for xylitol–sorbitol (E8/E11), and from 64.48 to 54.34 kJ mg^−1^ GAE for glycerol–xylitol (E9/E10). Overall, the more than three-fold variation in SEC among the investigated NADES-based systems shows that formulation composition can substantially affect energy efficiency when extraction conditions are standardized.

### 2.6. In Vitro Cytocompatibility of Neat NADES, NADES Extracts, and Cold-Pressed Oils

**Figure 7:**
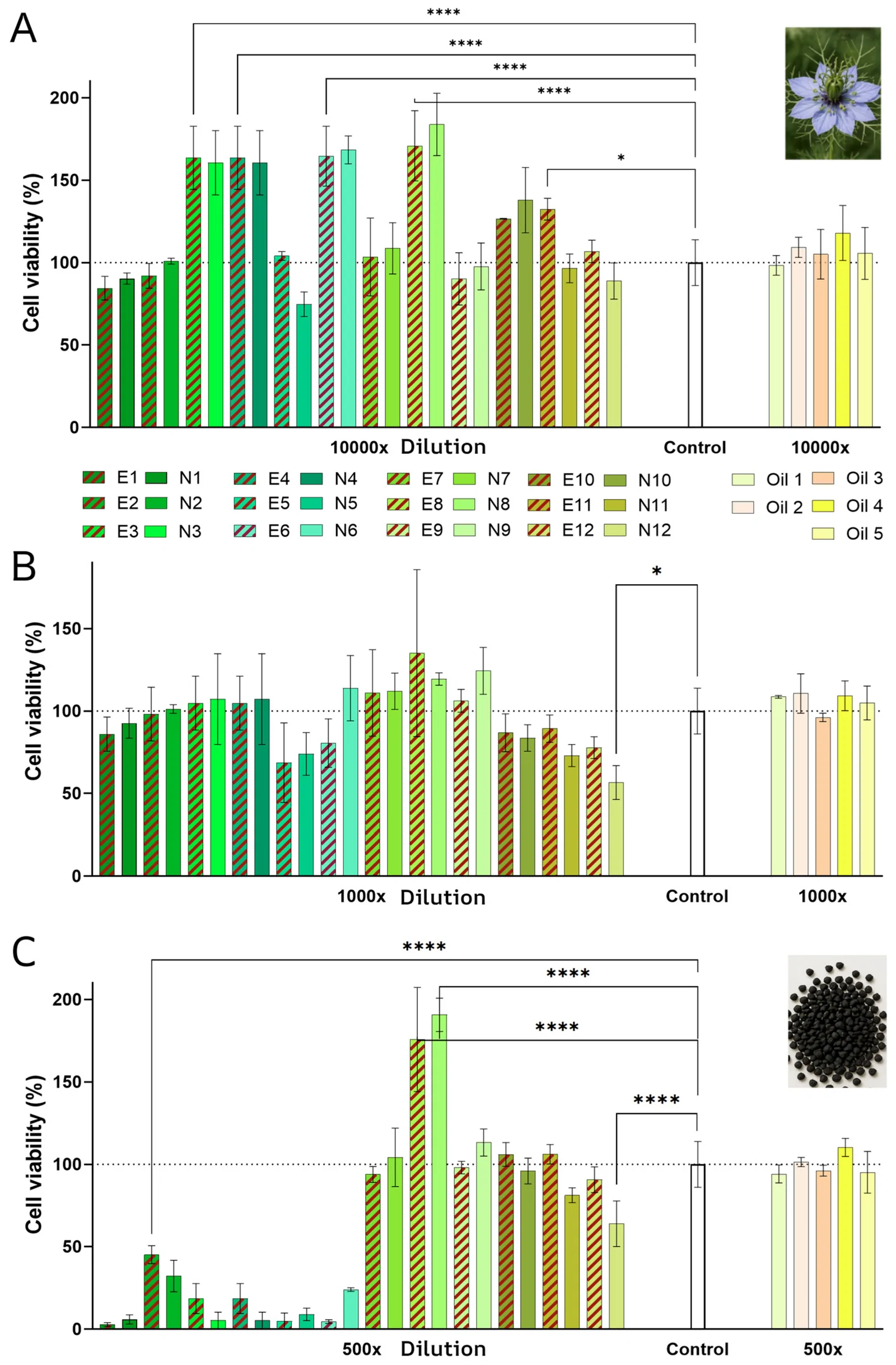
Dilution-dependent effects of neat NADES, their corresponding *Nigella sativa* extracts, and cold-pressed oils on HaCaT cell viability determined using the MTT assay. (**A**) 10,000×, (**B**) 1000×, and (**C**) 500× dilutions. Results are presented as mean ± SD relative to untreated control cells (100% viability). The dotted horizontal line indicates the viability of untreated control cells. Statistical significance was evaluated using one-way ANOVA followed by Dunnett’s multiple-comparison test (* *p* < 0.05; **** *p* < 0.0001).

To evaluate the cytocompatibility of the prepared NADES extracts and cold-pressed oils, HaCaT keratinocyte viability was assessed using the MTT assay. Dilution-dependent effects were examined at three dilutions (10,000×, 1000×, and 500×). NADES extracts were compared with the corresponding neat NADES tested at the same dilutions, while untreated cells served as the control (Figure 7).

At the highest dilution (10,000×; Figure 7A), most NADES formulations and their corresponding extracts remained cytocompatible. However, N5 reduced the mean cell viability to approximately 75%, indicating a moderate response that warranted attention despite remaining above the conventional 70% cytotoxicity threshold. Together with the very low apparent pH of the N5/E5 pair, this observation supported its selection for the subsequent partial-neutralization experiment. In contrast, E3, E4, E6, E8, and E11 produced MTT responses significantly above that of the untreated control. The corresponding neat formulations N3, N4, N6, and N8 exceeded 150% of the control response, while E10, N10, and E11 showed increases of approximately 30%. Because an increased MTT signal may reflect greater metabolic activity, a higher number of viable cells, or both, values above the untreated control were not interpreted as direct evidence of cell proliferation. In several cases (E5, E11, and E12), the extracts produced significantly higher MTT responses than their corresponding neat NADES formulations, indicating that the presence of extracted seed constituents modified the cellular response to these systems. The cold-pressed oils maintained cell viability close to control values.

At the intermediate dilution (1000×; Figure 7B), cell viability generally approached the untreated control. Most neat NADES formulations and their corresponding extracts remained non-cytotoxic. Lower responses were observed for N5/E5 and N12, with N12 reducing cell viability below 70% and showing the only statistically significant reduction relative to the untreated control.

At the lowest dilution (500×; Figure 7C), differences among the formulations became more pronounced. Neat NADES N1–N6 and their corresponding extracts E1–E6 reduced cell viability below 50%, indicating pronounced cytotoxicity at this dilution. In contrast, the N7/E7–N11/E11 systems remained close to or above the untreated control, and the N8/E8 pair produced the highest MTT response, reaching approximately 175–190% viability. N12 again deviated from this general pattern, reducing cell viability to approximately 65%, whereas E12 remained close to the control. Cold-pressed oils consistently maintained viability close to the untreated control throughout the dilution range investigated.

To examine whether the observed cytotoxicity was associated with solvent acidity, the highly acidic N5 and E5 formulations (apparent pH 0.28 and 0.35, respectively) were partially neutralized before testing. Partial pH adjustment restored cell viability at the 500× dilution, supporting a substantial contribution of acidity to the cytotoxic response of this formulation pair (Figure S5).

Collectively, these findings show that the biological response of the investigated systems depended strongly on formulation composition, dilution, and acidity. At the 10,000× dilution, most systems remained cytocompatible, although N5 produced a moderate reduction in cell viability that warranted further attention. Four of the five extracts with significantly elevated MTT responses contained sorbitol (E3, E6, E8, and E11), suggesting a likely association between sorbitol-containing formulations and increased MTT responses. Importantly, E5, E11, and E12 produced significantly higher MTT responses than their corresponding neat NADES formulations, demonstrating that the extracted seed constituents modified the cellular response relative to the parent solvents. At the 500× dilution, pronounced cytotoxicity was observed in the acid–polyol systems N1–N6/E1–E6, whereas the binary polyol and ternary citric acid–polyol systems generally retained higher viability, with N12 representing a notable exception. Partial neutralization of the highly acidic N5/E5 pair restored cell viability, supporting acidity as a major contributor to its cytotoxic response.

## 3. Discussion

Despite the extensive literature on *Nigella sativa*, research has been dominated by the composition, extraction, stability, and biological properties of its seed and fixed oils [1,9,32,33]. In contrast, the use of NADES for this species remains largely unexplored, with the few available studies addressing essential-oil recovery from seeds or antioxidant extraction from defatted seed cake [18,19]. Although NADES have been widely investigated for the recovery of plant bioactive compounds [14,15,16,17], to the best of our knowledge, no previous study has systematically employed edible NADES for the direct recovery of phenolic and antioxidant constituents from whole *Nigella sativa* seeds while retaining the extraction medium as part of a ready-to-use formulation. This shift from the extensively studied lipid fraction toward whole-seed extraction with edible NADES constitutes a central innovative aspect of the present work.

The present study shows that varying the composition of edible NADES changes not only their physicochemical behavior and extraction performance, but also the energy efficiency of the process and the cellular response to the resulting *Nigella sativa* extracts. By integrating physicochemical characterization with spectroscopic and thermal analyses, extraction performance, green processing metrics, and in vitro cytocompatibility, this work establishes a structure–property framework linking solvent composition with extraction outcomes.

In more detail, this study was based on the rational design of four compositional solvent families differing in organic acid and polyol composition, while maintaining standardized extraction conditions. Unlike salt-based DES, in which components are commonly assigned distinct hydrogen-bond acceptor and donor roles [22,34], the organic acids and polyols used here form a more distributed hydrogen-bonding network through their multiple hydroxyl and carboxyl groups [12,23,29]. The availability of several hydrogen-bonding sites allows their relative donor–acceptor contributions to vary with composition and local molecular environment. By applying the same extraction conditions to all formulations, the experimental design enabled the influence of solvent composition to be examined without confounding changes in extraction technology. This distinction is particularly important because NADES behavior depends not only on component identity but also on the molar ratio and water content, which together shape the hydrogen-bonding network, viscosity, polarity, acidity, and mass-transfer characteristics [21,35].

Comparison of the four deliberately constructed solvent families showed that component effects were context-dependent rather than additive. Within the malic acid series, viscosity increased in the order glycerol < xylitol < sorbitol, whereas the corresponding citric acid formulations occupied a narrower range, indicating that the influence of polyol identity depended on the accompanying organic acid. The ternary formulations were less viscous than the corresponding binary polyol systems; however, because these systems simultaneously differed in water content, citric acid proportion, H_2_O:NWC molar ratio, and overall hydrogen-bonding organization, the observed reduction in viscosity cannot be attributed to any single compositional factor. Similarly, the attenuation of the H_2_O:NWC–viscosity association after inclusion of the ternary formulations indicates that the relationship observed within the binary systems reflects the combined influence of formulation composition rather than an independent effect of the hydration descriptor alone. In the present dataset, the H_2_O:NWC molar ratio was used as a formulation-level hydration descriptor, not as an isolated predictor separable from component identity, molar composition, or hydrogen-bonding capacity. These observations further emphasize that water content, organic composition, and hydrogen-bonding organization must be interpreted jointly.

Water was incorporated as an integral component of all NADES formulations because controlled hydration reduces viscosity and facilitates mass transfer, while its molar proportion relative to the NWC influences the resulting hydrogen-bonding environment [29,35]. However, water alone was not included as a reference extraction solvent because it provides limited recovery of relatively lipophilic constituents, including thymoquinone, and thymoquinone undergoes rapid degradation in aqueous media [30]. Ethanol was therefore selected as the conventional reference solvent because it is food-compatible and provides a practical compromise for recovering chemically diverse constituents from *Nigella sativa* seeds. Methanol, although frequently effective for analytical extraction of phenolic compounds and thymoquinone [36], was not considered appropriate for comparison with edible NADES intended to remain in ready-to-use formulations [37].

Cold-pressed oils provided a practically relevant comparator, representing a widely consumed and topically applied *Nigella sativa* product obtained without solvent extraction. Their substantially lower TPC and ABTS values reflect the different selectivity and composition of the lipid matrix and should not be interpreted simply as inferior product quality. The considerable variability in oil-specific antioxidant assays and oxidative quality indices, despite broadly similar major fatty acid profiles in the two oils examined by GC-FID, is more plausibly related to differences in seed storage conditions [9], processing, and minor constituents [33,38]. Accordingly, the oil data contextualizes the NADES and ethanolic extracts without constituting the principal novelty of the present study.

The spectroscopic characterization performed in the present study further supports the rationale underlying the solvent-family design. ATR-FTIR analysis revealed reproducible family-related spectral patterns, particularly in the O–H stretching and fingerprint regions, consistent with differences in the hydrogen-bonding environments created by the selected acid–polyol combinations. Importantly, both the neat NADES formulations and their corresponding extracts retained their principal absorption bands after 15 days of storage at room temperature and under refrigeration, with only minor variations in relative band intensity. The absence of substantial band displacement indicates that the dominant intermolecular interaction patterns remained spectrally stable under both storage conditions. The observed broadening and shifts in the O–H stretching bands are consistent with the extensive and hydration-dependent intermolecular hydrogen bonding commonly reported for NADES [12,23,28,29,39]. Thus, the principal finding of the ATR-FTIR storage analysis is that the designed NADES and their extracts maintained their characteristic spectral profiles for at least 15 days, including at room temperature.

Because of this demonstrated spectral stability, DSC analysis was performed using the neat NADES and corresponding extracts from the 15-day refrigerated series, thereby providing a common and well-defined storage condition for thermal comparison. DSC distinguished the neat NADES from their corresponding extracts more clearly than ATR-FTIR. Whereas the vibrational spectra were dominated by the NADES matrix and showed only limited changes following extraction, the thermograms revealed formulation-specific redistribution in the number, position, and relative prominence of the recorded thermal events. These changes are consistent with modification of the thermal behavior of the systems following the incorporation of seed-derived constituents and their interactions with the solvent matrix. Because several events were broad, asymmetric, or overlapping, and the measurements covered the range from −20 to 300 °C, they were interpreted as thermal profiles rather than being assigned categorically to glass transitions, melting, crystallization, or decomposition. Thus, ATR-FTIR was particularly informative for evaluating the preservation of the dominant molecular interaction pattern, whereas DSC was more sensitive to extraction-associated changes in the overall thermal response [28,29,39].

The extraction results further show why NADES should not be treated as a uniformly superior solvent class. E1 produced the highest TPC and significantly exceeded both ethanolic extraction methods, whereas E8 produced the lowest TPC among the NADES extracts and was significantly below the Soxhlet extract. A different ranking emerged for ABTS activity: E9 was highest, followed by E1, and E1, E9, E10, and E12 significantly exceeded the Soxhlet extract. The weak-to-moderate TPC–ABTS association (ρ = 0.33) indicates that recovery of a larger total amount of phenolic-reactive material did not necessarily yield proportionally greater antioxidant activity. Instead, the response likely also depended on the identity, relative abundance, and individual activity of the extracted constituents, which explains why E1 maximized TPC while E9 maximized ABTS activity.

The formulation-dependent differences in extraction efficiency confirmed that NADES performance could not be explained by a single physicochemical parameter [14,16,20,40]. For the neat NADES, TPC was inversely associated with apparent pH (ρ = −0.67) and viscosity (ρ = −0.58), while ABTS activity was inversely associated with viscosity (ρ = −0.68) and density (ρ = −0.59). These relationships indicate that phenolic recovery and antioxidant activity were influenced by both solvent acidity and transport-related constraints. The inverse associations with viscosity are consistent with previous reports that high NADES viscosity restricts molecular diffusion and mass transfer from plant matrices [16,40,41]. Nevertheless, viscosity alone did not determine extraction performance, as formulations with comparable viscosity produced different TPC and ABTS values. This confirms that solvent–solute affinity, hydrogen-bonding interactions, and the combined effects of solvent composition must also be considered [13,20,42].

Comparison of the two correlation matrices provided further insight into when these relationships were established. After extraction, the correlations of TPC with apparent pH and viscosity weakened from −0.67 to −0.61 and from −0.58 to −0.52, respectively, while the ABTS–density correlation decreased markedly from −0.59 to −0.22. In contrast, the inverse association between ABTS activity and viscosity remained strong and increased slightly from −0.68 in the neat NADES to −0.73 in the corresponding extracts. Thus, the neat-solvent properties showed more consistent associations with extraction performance overall, but this pattern was not universal for every parameter. These findings suggest that the initial NADES composition establishes the physicochemical environment governing mass transfer and solute partitioning, whereas the properties measured after extraction additionally reflect the recovered seed constituents and their interactions with the solvent matrix. Accordingly, rational control of solvent composition, apparent pH, viscosity, and water content may reduce empirical trial-and-error by prioritizing formulations with properties associated with improved phenolic recovery and antioxidant performance.

The SEC analysis provides a process-level expression of the same formulation dependence. Because all ultrasound-assisted extractions received the same estimated energy input, their different SEC values arose directly from differences in recovered GAE under standardized conditions. Within both acid–polyol families, SEC increased consistently from glycerol- to xylitol- and sorbitol-containing formulations, paralleling the viscosity trend most clearly observed in the malic acid series. Among the binary polyol systems, the glycerol–xylitol formulation was more energy efficient than glycerol–sorbitol, while xylitol–sorbitol showed the highest SEC. The corresponding ternary formulations containing 0.1 mol citric acid exhibited a lower SEC in every matched comparison, most prominently for the glycerol–sorbitol and xylitol–sorbitol pairs. Because these ternary formulations also contained more water, the improvement is best interpreted as the consequence of the complete ternary formulation—combining altered acidity, viscosity, hydration, and solvent–solute interactions—rather than as an isolated citric acid effect.

Only E1, E4, E10, and E12 achieved a lower SEC than the Soxhlet benchmark. Conversely, ultrasound-assisted ethanolic extraction required substantially less total process energy than Soxhlet extraction but recovered insufficient GAE to achieve a lower SEC. These findings demonstrate that a shorter or nominally less energy-intensive process is not necessarily more efficient per unit of recovered target, and that replacing a conventional solvent with NADES does not automatically confer a sustainability advantage. Meaningful process improvement therefore requires simultaneous optimization of solvent composition, extraction yield, and operating energy rather than reliance on the green origin of the solvent constituents alone [41,42,43].

The HaCaT MTT assay further demonstrated that biological response was both dilution- and composition-dependent. At the 10,000× dilution, several neat NADES and extracts produced MTT signals above the untreated control; however, this increase may reflect greater metabolic activity, a higher number of viable cells, or both, and was therefore not interpreted as direct evidence of proliferation. At the 500× dilution, the acid–polyol systems N1–N6 and E1–E6 markedly reduced viability, whereas most binary polyol and ternary systems remained close to or above the control, with N12 as a notable exception. In contrast, the cold-pressed oils consistently maintained cell viability close to the untreated control across all three tested dilutions. The recurring elevated responses among sorbitol-containing extracts are an interesting compositional association, but the present data do not establish a component-specific causal effect. Partial neutralization restored viability for N5/E5, demonstrating that acidity contributed substantially to the response of this pair, without implying that acidity alone explains the behavior of every formulation [44]. Accordingly, the MTT assay was designed to compare the cytocompatibility of the investigated formulations and extracts relative to untreated cells and the corresponding neat NADES formulations, rather than to benchmark their effects against a known cytotoxic agent. Since a dedicated positive cytotoxicity control was not included, these findings are interpreted as relative differences in cell viability among the investigated formulations and tested dilutions.

Taken together, the results define an application-dependent design problem rather than a single universally optimal NADES. E1 combined the highest phenolic recovery with the lowest SEC, E9 provided the greatest ABTS activity, and several formulations showed favorable cytocompatibility only within an appropriate dilution range. Thus, ready-to-use potential must be evaluated against the intended route of use, required bioactive profile, acceptable acidity and viscosity, and biologically compatible concentration. By linking these endpoints within a common experimental framework, the present study moves beyond empirical solvent screening and provides a rational basis for selecting edible NADES formulations for sustainable recovery and delivery of *Nigella sativa* bioactives.

## 4. Materials and Methods

### 4.1. Chemicals and Reagents

All chemicals and reagents were of analytical grade and used without further purification. Glycerol was obtained from ZORKA Pharma (Šabac, Serbia), xylitol from Thermo Fisher Scientific (Waltham, MA, USA), and thiazolyl blue tetrazolium bromide (MTT), dimethyl sulfoxide (DMSO), sorbitol, and L-malic acid from Sigma-Aldrich (Darmstadt, Germany). Food-grade citric acid (Centroproizvod, Serbia) was purchased from a local market. Ultrapure water used for NADES preparation was produced using a Barnstead™ Smart2Pure™ water purification system (Thermo Fisher Scientific, Waltham, MA, USA).

Absolute ethanol, Folin–Ciocalteu reagent, sodium carbonate, potassium persulfate, potassium hydroxide, hydrochloric acid, phenolphthalein, glacial acetic acid, and Hanus solution were purchased from Merck/Supelco (Merck KGaA, Darmstadt, Germany). DPPH was obtained from Fluka (Buchs, Switzerland), whereas ABTS, Trolox, and gallic acid were supplied by Sigma-Aldrich (Darmstadt, Germany). For cell culture experiments, Dulbecco’s modified Eagle medium (DMEM), fetal bovine serum (FBS), penicillin–streptomycin, and trypsin–EDTA were obtained from Gibco (Thermo Fisher Scientific, Waltham, MA, USA).

### 4.2. Black Cumin Plant Material and Cold-Pressed Oils

Mature seeds of black cumin (*Nigella sativa* L.), originating from Egypt and harvested in the 2025 season, were obtained from Porta Naturae d.o.o., Srebrenik, Bosnia and Herzegovina. Upon receipt, seeds were visually inspected to remove impurities, damaged seeds, and foreign admixtures, and stored in tightly closed containers, protected from light and moisture at room temperature until use. In addition, five cold-pressed *Nigella sativa* oils were included for comparative evaluation. One was produced in-house using a laboratory-scale cold-press under controlled conditions, whereas four were purchased from the local market (commercial cold-pressed oils). To avoid commercial bias, the identities of the manufacturers are not disclosed, and the oils are referred to throughout the manuscript as Oil 1–5.

### 4.3. Preparation of NADES

NADES were prepared using a heating and stirring method. The individual components were accurately weighed according to the formulation-specific molar ratios presented in Table 1. In the ternary formulations N10–N12, the citric acid proportion was reduced to 0.1 mol relative to each polyol to obtain less acidic formulations while retaining citric acid as a functional constituent of the hydrogen-bonding network. Water was incorporated as an integral formulation component at 25 wt% in N1–N9 and 30 wt% in N10–N12, expressed relative to the total mass of the final formulation. For complete compositional description, water was additionally expressed as the H_2_O to NWC molar ratio, calculated as the number of moles of water divided by the total number of moles of all non-water components. The mixtures were then stirred continuously at 320 rpm for 1 h using a magnetic hotplate stirrer (LLG-uniSTIRRER 3, LLG Labware, Meckenheim, Germany) in a water bath maintained at 65–70 °C until clear and homogeneous solvents were obtained.

### 4.4. Ultrasound-Assisted Extraction of Nigella sativa Seeds Using NADES and Ethanol

Prior to extraction, the seed material was ground using the NutriBullet blender (De’Longhi Group, Los Angeles, CA, USA) in three cycles of 3 s each, producing particles with an average diameter of approximately 1.5 mm. For extraction, 4 g of seed material was combined with the respective solvents at a solid-to-solvent ratio of 1:10 (*w*/*v*) in the Erlenmeyer flask (50 mL). UAE was then performed in an ultrasonic water bath (Elmasonic P, Elma Schmidbauer, GmbH, Singen, Germany) for 30 min at a frequency of 37 kHz and 60% power (192 W). The temperature was carefully monitored and did not exceed 40 °C during the process. To prevent solvent loss, the Erlenmeyer flasks were sealed with parafilm throughout sonication. These conditions facilitated efficient mass transfer and homogenization.

The maximum theoretical energy input per mL upon sonication was estimated using the equation:

(1) $$E\;=\;\frac{P\cdot t}{V}\;=\;\frac{192\;W\cdot 1800\;s}{40\;\mathrm{mL}}\;=\;8.64\;\mathrm{kJ}/\mathrm{mL}$$

where *E* is the energy density (J/mL), *P* is the ultrasound power (W), *t* is the sonication time (s), and *V* is the sample volume (mL). The calculated value represents the maximal possible (theoretical) energy input, since the actual delivered energy is typically lower due to cavitation efficiency and heat losses in the ultrasonic bath.

After sonication, the extracts were transferred into 50 mL Falcon tubes, centrifuged for 15 min at 4500 rpm, and the supernatant was collected using an automatic pipette. One series was stored refrigerated, another frozen, and the third kept at room temperature (20 °C) in the dark.

### 4.5. Soxhlet Extraction of Nigella sativa Seeds with Ethanol

Soxhlet extraction was performed using a 500 mL Soxhlet apparatus connected to a 1 L round-bottom flask. A total of 30 g of ground *Nigella sativa* seeds were placed in a cellulose extraction thimble, and 300 mL of HPLC-grade absolute ethanol was used as the solvent. The extraction was carried out under continuous reflux for 4 h, achieving approximately 16–20 complete siphon cycles. The maximum theoretical energy input per solvent mL upon extraction was 9.60 kJ/mL. Upon completion, the liquid ethanolic extract was cooled to room temperature and stored in a tightly closed amber glass container at 4 °C, protected from light and moisture, until further analysis.

### 4.6. Determination of Apparent pH, Viscosity, Density and Refractive Index of Extracts and Oils

The apparent pH of neat NADES and the corresponding NADES-based and ethanolic extracts were measured using a FiveEasy pH meter (Mettler Toledo, Greifensee, Switzerland). Given the solvent-dependent electrode response in these mixed aqueous and ethanolic media, the obtained values were treated as operational apparent pH measurements and used for comparative purposes rather than interpreted as conventional aqueous pH.

The dynamic and kinematic viscosities of the liquid samples were determined using an Ostwald viscometer at a controlled temperature of 22.0 °C. The density (ρ) of each sample was precisely measured using a standard 10.0000 mL glass pycnometer. Mass measurements were performed to the nearest 0.01 mg using a dual-range semi-micro analytical balance (AS 82/220, Radwag, Radom, Poland). Distilled water was used as the reference standard to calibrate the viscometer, exhibiting a reference density of 0.99777 g/mL and a dynamic viscosity of 0.5440 mPa·s at the testing temperature. The flow times for both the reference water (t_H2O_) and the samples (t_sample_) were recorded in triplicate to ensure reproducibility. The dynamic viscosity (η) was calculated using the relative viscometry equation based on the ratio of densities and flow times, while the kinematic viscosity (ν) was derived by dividing the dynamic viscosity by the respective sample density. The refractive indices (n_D_) of the NADES were determined at a controlled temperature of 22.0 °C using a high-precision digital refractometer. Before the measurements, the instrument was calibrated with distilled water, and the prism surface was thoroughly cleaned between consecutive samples to prevent cross-contamination.

### 4.7. Characterization of Cold-Pressed Nigella sativa Oils

The cold-pressed oils were characterized in terms of their physicochemical properties, moisture and water contents, oxidative quality indices, DPPH radical-scavenging activity, ferric reducing antioxidant power (FRAP), and fatty acid composition. Fatty acid profiles of the selected oils were determined as fatty acid methyl esters by gas chromatography with flame-ionization detection (GC-FID). Detailed analytical procedures are provided in Supplementary Materials Sections S1.1–S1.3.

### 4.8. ATR-FTIR Characterization and Storage Stability of Pure NADES and Extracts

Attenuated Total Reflectance–Fourier Transform Infrared Spectroscopy (ATR-FTIR) spectra of the solid precursors, pure NADES, and *Nigella sativa* seed extracts (NADES-based and ethanolic) were recorded using a Nicolet iS20 spectrometer with a Smart iTX Diamond ATR accessory (Thermo Fisher Scientific Inc., Waltham, MA, USA) and OMNIC Paradigm software, version 2.7 (4000–650 cm^−1^ range, 4 cm^−1^ resolution, 16 scans, air background baseline correction).

Pure crystalline solid components (e.g., citric and malic acids) were placed directly onto the crystal and firmly compressed using the built-in high-pressure anvil to ensure optimal optical contact. For liquid NADES and NADES-based extracts, a few drops were applied directly onto the crystal and analyzed immediately at ambient temperature. For ethanolic extracts, spectra were recorded after complete solvent evaporation from the crystal, leaving a dry film.

A systematic screening was conducted to evaluate the temporal and thermal stability of both NADES systems and *Nigella sativa* extracts. For pure NADES, ATR-FTIR spectra were recorded immediately after synthesis (1–2 h) and again after a 24 h stabilization period. For the extracts, spectra were collected directly after extraction (day 0). To assess longer-term stability, samples were stored at room temperature (20 °C, dark) and under refrigeration (4 °C). After 15 days, both NADES and extract series from each storage condition were analyzed by ATR-FTIR to identify potential changes in hydrogen bonding networks and chemical cohesion.

### 4.9. DSC Characterization of Pure NADES and Nigella sativa Extracts

The thermal properties and phase transition behaviors of the neat NADES vehicles and their corresponding *Nigella sativa* seed extracts were analyzed via differential scanning calorimetry using a DSC131 Evo thermal analyzer (SETARAM Instrumentation, KEP Technologies, Caluire-et-Cuire, France). Samples (approximately 5–15 mg) of the liquid NADES and NADES-based extracts were precisely introduced into 120 μL aluminum pans and immediately hermetically sealed, utilizing an empty, sealed aluminum pan as the reference control.

The experimental sequence was initiated by pre-cooling both the reference and sample crucibles at −20 °C for 10 min to establish thermal equilibrium. Subsequently, the pans were subjected to a controlled dynamic heating ramp from −20 °C up to a maximum temperature of 300 °C at a constant heating rate of 10 °C/min. An inert atmosphere was continuously maintained throughout the thermal runs under a steady nitrogen purge at a flow rate of 20 mL/min.

To ensure baseline stability, an instrumental baseline run was performed using empty crucibles under identical experimental conditions. Baseline automated subtraction and transition enthalpy determinations (J/g) were executed using the integrated CALISTO PROCESSING software version 2.0 (SETARAM Instrumentation, Caluire-et-Cuire, France). All reported thermodynamic values represent the mean of three independent measurements.

### 4.10. Total Phenolic Content (TPC)

The total phenolic content (TPC) of *Nigella sativa* extracts and oils was determined spectrophotometrically using the Folin–Ciocalteu method adapted to a 96-well microplate format, as previously described [44], with further modifications.

To eliminate interference from reagent background and inherent sample coloration, a dual-blanking protocol was applied: reagent blanks (sample replaced with extraction vehicle) and sample blanks (Folin–Ciocalteu replaced with water).

Calibration curves of gallic acid (25–500 µg/mL) were generated in three different matrices to account for the strong influence of medium acidity on chromophore development: ethanol (neutral reference), N4 NADES (citric acid-based, pH 0.12, strongly acidic), and N8 NADES (polyol mixture, pH ≈ 3, moderately acidic). Each extract was quantified against its corresponding matrix-matched calibration curve.

In a typical layout, wells received 10 µL of sample or standard, 10 µL Folin–Ciocalteu reagent (or water for sample blanks), followed by 6 min incubation at room temperature. Then, 100 µL of 7.5% sodium carbonate solution and 60 µL distilled water were added to a final volume of 180 µL per well. Reaction mixtures were incubated in the dark for 90 min before measuring absorbance at 760 nm (Synergy LX Multimode Microplate Reader, BioTek Instruments, Winooski, VT, USA).

For comparative purposes, all TPC and ABTS values, including those obtained for cold-pressed oils, ethanolic extracts, and NADES-based extracts, were ultimately expressed on a seed mass basis (per g of seed). For oil samples, the measured values were corrected for the dilution factor, converted using oil density, and normalized using the average oil yield obtained from the seeds. Final absorbance values were corrected by subtracting both reagent and sample blanks. All measurements were performed in quadruplicate. TPC values were expressed uniformly as milligrams of gallic acid equivalents per gram of seed (mg GAE/g seed).

### 4.11. Antioxidant Capacity Assays (ABTS)

The antioxidant capacity of NADES-based extracts (E1–E12), ethanolic extracts (E13–E14), and cold-pressed oil samples was determined using the ABTS•+ radical cation assay of Re et al., [45], adapted to a 96-well microplate format, as previously described by Smiljanić et al., [44], with further modifications.

The ABTS•+ chromophore was prepared by mixing 7 mM ABTS with 2.45 mM potassium persulfate in water and incubating in the dark at room temperature for 12–16 h.

Each well contained 10 µL of sample or standard and 190 µL of ABTS•+ solution, followed by incubation for 30 min at room temperature in the dark. Absorbance was measured at 734 nm using a Synergy LX Multimode Microplate Reader (BioTek Instruments, Winooski, VT, USA). Trolox calibration curves (25–600 µM) were prepared in MeOH reagent blanks (10 µL MeOH + 190 µL ABTS•+) and NADES-specific blanks prepared with neat NADES vehicles. Neat NADES solutions were also tested as negative controls. Sample blanks (extract/oil dilutions without radical) were included to correct for background absorbance. All measurements were performed in quadruplicate.

The percentage of radical decolorization (% inhibition) was calculated as:

$$\%\;\mathrm{Decolorization}=\left(1-\frac{A_{\mathrm{sample}}-A_{\mathrm{sample}\;\mathrm{blank}}}{A_{\mathrm{reagent}\;\mathrm{blank}}}\right)\times 100$$

where $A_{\mathrm{sample}}$ is the absorbance of the reaction mixture, $A_{\mathrm{sample}\;\mathrm{blank}}$ the absorbance of the sample without radical, and $A_{\mathrm{reagent}\;\mathrm{blank}}$ the absorbance of the ABTS•+ solution. Radical scavenging capacity was derived from Trolox calibration curves, corrected for dilution, and expressed as µmol Trolox equivalents per gram of seed (µmol TE/g seed).

### 4.12. Cytotoxicity Assay

#### 4.12.1. Cell Culture

Human keratinocytes (HaCaT; AddexBio, No. T0020001) were maintained as monolayers in DMEM. The medium was supplemented with 1% non-essential amino acids (NEAA), 100 IU/mL penicillin, 0.1 mg/mL streptomycin, 2 mM glutamine, 1 mM sodium pyruvate, and 10% FBS. Cultures were incubated at 37 °C in a humidified atmosphere containing 5% CO_2_.

#### 4.12.2. Assessment of Cell Viability

HaCaT cells were seeded at 10,000 cells per well in 96-well microtiter plates. After 24 h of adhesion, *Nigella sativa* NADES extracts and cold-pressed oils were added at final dilutions of 10,000×, 1000× and 500× in DMEM. Control wells received only nutrient medium. A dedicated positive cytotoxicity control was not included. All experiments were performed in triplicate. Cell viability was determined using the MTT assay after 24 h of treatment, following a modified Mosmann protocol [46]. Briefly, 20 µL of MTT solution (5 mg/mL in phosphate-buffered saline, PBS) was added to each well and incubated for 2 h at 37 °C in 5% CO_2_. The medium was then aspirated, and 200 µL of DMSO was added to solubilize the formazan crystals. Absorbance was measured at 570 nm (formazan signal) and 630 nm (background), and viability was calculated as the corrected value A570–A630 using a BioTek Synergy LX spectrophotometer (Agilent Technologies, Santa Clara, CA, USA). Results were expressed as percentage viability, with untreated cells set to 100%.

### 4.13. Statistical Analysis and Graph Creation

All experimental measurements were performed in triplicate unless otherwise stated, and the results are expressed as means ± standard deviations (SD). Statistical analyses were performed using GraphPad Prism, version 11.02. Differences among groups were evaluated by one-way analysis of variance (ANOVA), followed by Sidak’s or Dunnett’s multiple comparisons test, as specified in the corresponding figure legends, with statistical significance accepted at *p* < 0.05. Relationships between physicochemical properties and bioactivity parameters were assessed using Spearman’s rank correlation coefficient (ρ), and correlation matrices were generated in GraphPad Prism, version 11.02. Graphs were prepared using GraphPad Prism and Microsoft Excel, while figures and graphical layouts were assembled using the web-based Photopea image editor (https://www.photopea.com/).

## Figures and Tables

**Table 1:** Composition and physicochemical properties of the designed edible NADES formulations.

NADES	Composition	Molar Ratio of Non-Water Components (NWC)	Water		Apparent pH	Density (g/mL)	Dynamic Viscosity, η (mPa·s)	Refractive Index ($n_{D}^{22}$)
			H_2_O:NWC (mol/mol)	(wt%)				
N1	Gly:MA	1:1	2.1:1	25	0.18	1.268 ± 0.001	15.07 ± 0.54	1.4366
N2	Xyl:MA	1:1	2.6:1	25	0.51	1.289 ± 0.002	41.41 ± 0.42	1.4516
N3	Sor:MA	1:1	2.9:1	25	0.51	1.301 ± 0.002	83.67 ± 1.20	1.4557
N4	Gly:CA	1:1	2.6:1	25	0.12	1.277 ± 0.001	51.33 ± 0.41	1.4466
N5	Xyl:CA	1:1	3.2:1	25	0.28	1.306 ± 0.004	69.37 ± 1.15	1.4557
N6	Sor:CA	1:1	3.5:1	25	0.35	1.298 ± 0.003	68.96 ± 0.16	1.4557
N7	Sor:Gly	1:1.2	2.5:1	25	2.38	1.248 ± 0.004	53.24 ± 0.53	1.4547
N8	Xyl: Sor	1:1	3.1:1	25	3.00	1.285 ± 0.002	94.18 ± 0.42	1.4667
N9	Gly:Xyl	1:1	2.3:1	25	4.52	1.188 ± 0.001	19.00 ± 0.15	1.4396
N10	CA:Gly:Xyl	0.1:1:1	3.0:1	30	1.78	1.215 ± 0.005	17.45 ± 0.39	1.4406
N11	CA:Xyl:Sor	0.1:1:1	4.0:1	30	1.86	1.266 ± 0.003	34.39 ± 0.54	1.4557
N12	CA:Sor:Gly	0.1:1:1	3.3:1	30	1.76	1.227 ± 0.004	30.21 ± 0.26	1.4476

Density and dynamic viscosity data are presented as means ± standard deviations (*n* = 3). Apparent pH values were obtained by direct potentiometric measurement with an accuracy of ±0.01 and are intended for comparative interpretation rather than as conventional aqueous pH values. The refractive index was measured at 22 °C with an accuracy of ±0.0001. The H_2_O:NWC molar ratio represents the number of moles of water relative to one mole of total non-water components. Water content (wt%) denotes the mass fraction of water in the final formulation. Gly, glycerol; Xyl, xylitol; Sor, sorbitol; MA, malic acid; CA, citric acid; NWC, non-water components.

**Table 2:** Summary of the principal DSC profile changes observed between the designed NADES formulations and their corresponding *Nigella sativa* extracts.

Pair	Composition	Number of Annotated Events (NADES/Extract)	Dominant *T*_peak_: NADES → Extract, °C (Δ*T*_peak_)	Overall DSC Profile Response After Extraction
N1/E1	MA + glycerol (1:1)	3/2	150.2 → 128.0 (−22.2)	Simplified profile; dominant response redistributed to a lower temperature
N2/E2	MA + xylitol (1:1)	3/3	127.8 → 141.9 (+14.1)	Event count unchanged; dominant response occurred at a higher temperature
N3/E3	MA + sorbitol (1:1)	3/6	189.4 → 204.1 (+14.7)	Markedly increased profile complexity; dominant response occurred at a higher temperature
N4/E4	CA + glycerol (1:1)	3/3	190.6 → 70.3 (−120.3)	Major redistribution of the dominant response to a lower temperature
N5/E5	CA + xylitol (1:1)	3/2	182.5 → 182.4 (−0.1)	Dominant response preserved; low-temperature profile simplified
N6/E6	CA + sorbitol (1:1)	2/4	173.7 → 182.4 (+8.7)	Increased profile complexity; dominant response occurred at a slightly higher temperature
N7/E7	sorbitol + glycerol (1:1.2)	2/4	118.6 → 140.7 (+22.1)	Increased profile complexity; dominant response occurred at a higher temperature
N8/E8	xylitol + sorbitol (1:1)	3/2	134.5 → 121.6 (−12.9)	Simplified profile; dominant response occurred at a lower temperature
N9/E9	glycerol + xylitol (1:1)	4/4	196.4 → 130.8 (−65.6)	Event count unchanged; dominant response redistributed to a lower temperature
N10/E10	CA + glycerol + xylitol (0.1:1:1)	2/3	130.1 → 133.5 (+3.4)	Dominant response preserved; additional lower-temperature events resolved
N11/E11	CA + xylitol + sorbitol (0.1:1:1)	2/3	92.3 → 126.8 (+34.5)	Increased profile complexity; dominant response occurred at a higher temperature
N12/E12	CA + sorbitol + glycerol (0.1:1:1)	3/3	76.3 → 142.3 (+65.9)	Event count unchanged; dominant response redistributed to a higher temperature

*T*_peak_, temperature of the annotated peak maximum. The dominant event was operationally defined as the event with the largest absolute integrated heat within each thermogram (Table S2). Δ*T*_peak_ = extract dominant *T*_peak_ − neat-NADES dominant *T*_peak_. These descriptors summarize comparative thermal-profile changes and do not assign the events to specific physical processes.

## Data Availability

The original data supporting the findings of this study are openly available in Zenodo at https://doi.org/10.5281/zenodo.21721841. Additional data are provided within the article and its Supplementary Materials.

## Supplementary Materials

The following supporting information can be downloaded at: https://www.mdpi.com/article/10.3390/molecules31162854/s1, Figure S1: Association between the H_2_O molar ratio and dynamic viscosity of the designed NADES formulations; Figure S2: ATR-FTIR spectra of the pure starting components; Figure S3: ATR-FTIR spectra of neat NADES formulations N1–N12 under the investigated storage conditions; Figure S4: ATR-FTIR spectra of the designed NADES formulations and their corresponding *Nigella sativa* extracts; Figure S5: Effect of partial neutralization of N5 and E5 on HaCaT cell viability; Table S1: Apparent pH measurements of NADES, NADES-based extracts, and ethanolic extracts of *Nigella sativa*; Table S2: DSC integration intervals, peak temperatures, and integrated heat values of the neat NADES formulations and their corresponding *Nigella sativa* extracts; Table S3: Physicochemical quality, antioxidant capacity, and oxidative quality indices of the analyzed cold-pressed *Nigella sativa* oils; Table S4: Physicochemical properties of the in-house oil and Oil 3 before and after centrifugation; Table S5: Fatty acid composition of the in-house oil and Oil 3 before and after centrifugation determined by GC-FID; Table S6: Total phenolic content and ABTS antioxidant activity of the investigated ethanolic extracts, NADES-based extracts, and cold-pressed oils, with Šídák-adjusted pairwise comparisons against Soxhlet ethanolic extraction. References [47,48,49,50,51,52] are cited in the Supplementary Materials.

## Abbreviations

The following abbreviations are used in this manuscript:

ABTS	2,2′-azino-bis(3-ethylbenzothiazoline-6-sulfonic acid)
ATR-FTIR	attenuated total reflectance–Fourier-transform infrared spectroscopy
CA	citric acid
DMEM	Dulbecco’s modified Eagle medium
DMSO	dimethyl sulfoxide
DPPH	2,2-diphenyl-1-picrylhydrazyl
DSC	differential scanning calorimetry
FBS	fetal bovine serum
FRAP	ferric reducing antioxidant power
GAE	gallic acid equivalents
GC-FID	gas chromatography with flame ionization detection
Gly	glycerol
HaCaT	spontaneously immortalized human keratinocyte cell line
HBA	hydrogen-bond acceptor
HBD	hydrogen-bond donor
MA	malic acid
MTT	3-(4,5-dimethylthiazol-2-yl)-2,5-diphenyltetrazolium bromide
NADES	natural deep eutectic solvents
NEAA	non-essential amino acids
NWC	non-water components
PBS	phosphate-buffered saline
SEC	specific energy consumption
Sor	sorbitol
TE	Trolox equivalents
TPC	total phenolic content
UAE	ultrasound-assisted extraction
Xyl	xylitol
