# Supplementary material for: From Solvent Design to Biological Response: Structure–Property Relationships of Edible Natural Deep Eutectic Solvents for the Extraction of Nigella sativa Bioactives
Source: Molecules. 2026 Aug 15;31(16):2854. doi: 10.3390/molecules31162854 (PMC13515620; doi:10.3390/molecules31162854)
Supplement: Supplementary file 1 [file molecules-31-02854-s001.zip › molecules-4448421-supplementary.pdf]

molecules

Supplementary Materials

From Solvent Design to Biological Response: Structure–Property Relationships of Edible Natural Deep Eutectic Solvents for the Extraction of *Nigella sativa* Bioactives

Emina Mehmedović [1], Vesna Jovanović [2], Maja Krstić Ristivojević [2], Smilja Marković [3], Ivana Prodić [4], Husejin Keran [5], and Katarina Smiljanić [2,*]

[1] Faculty of Technology and Faculty of Natural Sciences and Mathematics, University of Tuzla, 75000 Tuzla, Bosnia and Herzegovina emiina.mehmedovic@gmail.com;
[2] CoE for Molecular Food Sciences, Department of Biochemistry, University of Belgrade—Faculty of Chemistry, Studentski trg 12-16, 11158 Belgrade, Serbia vjovanovic@chem.bg.ac.rs; krstic_maja@chem.bg.ac.rs; katarinas@chem.bg.ac.rs;
[3] Institute of Technical Sciences of SASA, Knez Mihailova 35/IV, Belgrade 11000, Serbia smilja.markovic@itn.sanu.ac.rs;
[4] Institute of Virology, Vaccines and Sera "Torlak"—National Institute of the Republic of Serbia, Vojvode Stepe 458, 11152 Belgrade, Serbia, iprodic@torlak.rs;
[5] Faculty of Technology, University of Tuzla, 75000 Tuzla, Bosnia and Herzegovina husejin.keran@untz.ba;
* Correspondence: katarinas@chem.bg.ac.rs;

1. Materials and Methods

1.1. Physicochemical Properties, Moisture Analysis, and Oxidative Quality Indices of Cold-Pressed Oils

The chemical quality indices of cold-pressed *Nigella sativa* seed oils were determined using established titrimetric and electrochemical methods. Free acidity was measured by titration of oil dissolved in ethanol: diethyl ether (1:1, v/v) with standardized NaOH, using phenolphthalein as indicator; potentiometric titration was applied for darker matrices. The p-anisidine value, used as an indicator of secondary lipid oxidation products, was determined spectrophotometrically after reaction of the oil solution in isooctane with p-anisidine reagent in glacial acetic acid, with absorbance measured at 350 nm [47]. The peroxide value was quantified iodometrically after reaction with KI and starch indicator, followed by titration with $Na_2S_2O_3$. The iodine value was determined by the Hanus method, involving treatment with iodine monobromide solution and subsequent titration with $Na_2S_2O_3$ [48]. The saponification value was obtained by refluxing oil with ethanolic KOH and back-titrating the excess alkali with HCl [49].

The refractive index (n_D) was recorded at 20.0 °C using a digital refractometer. Values were monitored both in crude oil ($n_D = 1.4723$) and after centrifugation ($n_D = 1.4725$). Moisture content was assessed by two complementary approaches: gravimetric drying at 103 ± 2 °C until constant mass (0.48%) and volumetric Karl Fischer titration with potentiometric endpoint detection (0.14%) [50].

All physicochemical determinations were performed in triplicate, except DPPH and moisture, which are reported as single derived values.

1.2. DPPH Radical-Scavenging Activity and Ferric Reducing Antioxidant Power (FRAP) of Cold-Pressed Oils

Concurrently, the antioxidant profile of the cold-pressed oil samples was further characterized using the DPPH radical scavenging and Ferric Reducing Antioxidant Power (FRAP) assays. For the DPPH assay, the oil samples were mixed with a methanolic DPPH radical solution, incubated in the dark, and the absorbance decrease was recorded at 517 nm. The FRAP assay was conducted by mixing the oil

molecules

MDPI

samples with freshly prepared FRAP reagent (containing 2,4,6-tripyridyl-*s*-triazine, ferric chloride, and acetate buffer), and the increase in absorbance was measured at 593 nm. Trolox was employed as the reference standard for all assays, and the results are expressed as Trolox equivalents (TE).

1.3. GC-FID Analysis of Fatty Acid Methyl Esters of Cold-Pressed Oils

The fatty acid profiles of the cold-pressed *Nigella sativa* seed oils, comprising the in-house extracted oil and a commercial sample (*Nigellus*, Srebrenik) were determined as fatty acid methyl esters (FAMEs) using gas chromatography coupled with a flame ionization detector (GC-FID).

Prior to chromatographic separation, a rapid, one-step transesterification was performed under ambient laboratory conditions. Briefly, 10 mg of each oil sample was thoroughly dissolved in 500 μL of tert-butyl methyl ether (TBME). Subsequently, the fatty acids were converted into their corresponding volatile methyl esters via the addition of a trimethyl sulfonium hydroxide (TMSH) solution, following a rapid derivatization protocol optimized for lipid matrices [51]. The prepared reaction mixtures were immediately transferred into clean chromatographic vials for automated analysis.

Chromatographic separation was executed on an Agilent 8860 GC System (Agilent Technologies, Santa Clara, CA, USA) equipped with an automatic liquid sampler (ALS) and a flame ionization detector (FID). High-resolution separation of the FAME compounds, including critical saturated and unsaturated fatty acid isomers, was achieved using a specialized CP-Sil 88 for FAME capillary column (100 m × 250 μm i.d., 0.20 μm film thickness; Agilent Technologies, USA). An injection volume of 0.5 μL was introduced via the front injector.

The oven temperature program was initiated at 80 °C with an initial hold time of 0.1 min, increased to 220 °C at a constant heating rate of 4 °C/min (held for 5 min), and finally ramped at 4 °C/min to a maximum temperature of 240 °C with a final isothermal hold of 13 min (total run time: 58.1 min). Identification of individual fatty acids was performed by comparing the retention times of the sample peaks with those of pure reference standards, and the results are expressed as the relative area percentage (%) of total fatty acids based on standard protocols for edible vegetable oils [52].

2. Results

2.1. Association between Water Molar Ratio and Dynamic Viscosity

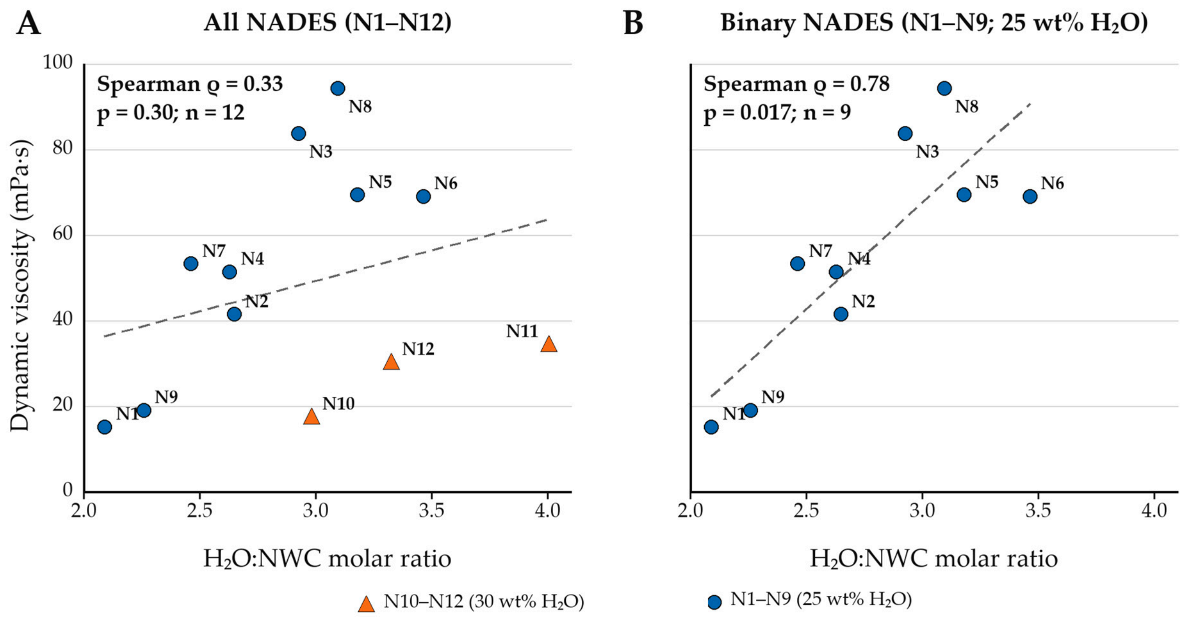

Figure S1. Association between the H_2O molar ratio and dynamic viscosity of the designed NADES formulations. (A) Analysis including all twelve formulations. (B) Analysis restricted to the binary formulations N1–N9, containing a constant water content of 25 wt%. Spearman's rank-correlation coefficients (ϱ) and corresponding *p*-values are displayed in each panel. NWC, non-water components.

2.2. *ATR-FTIR Characterization and Storage Stability*

Start of Figure S2

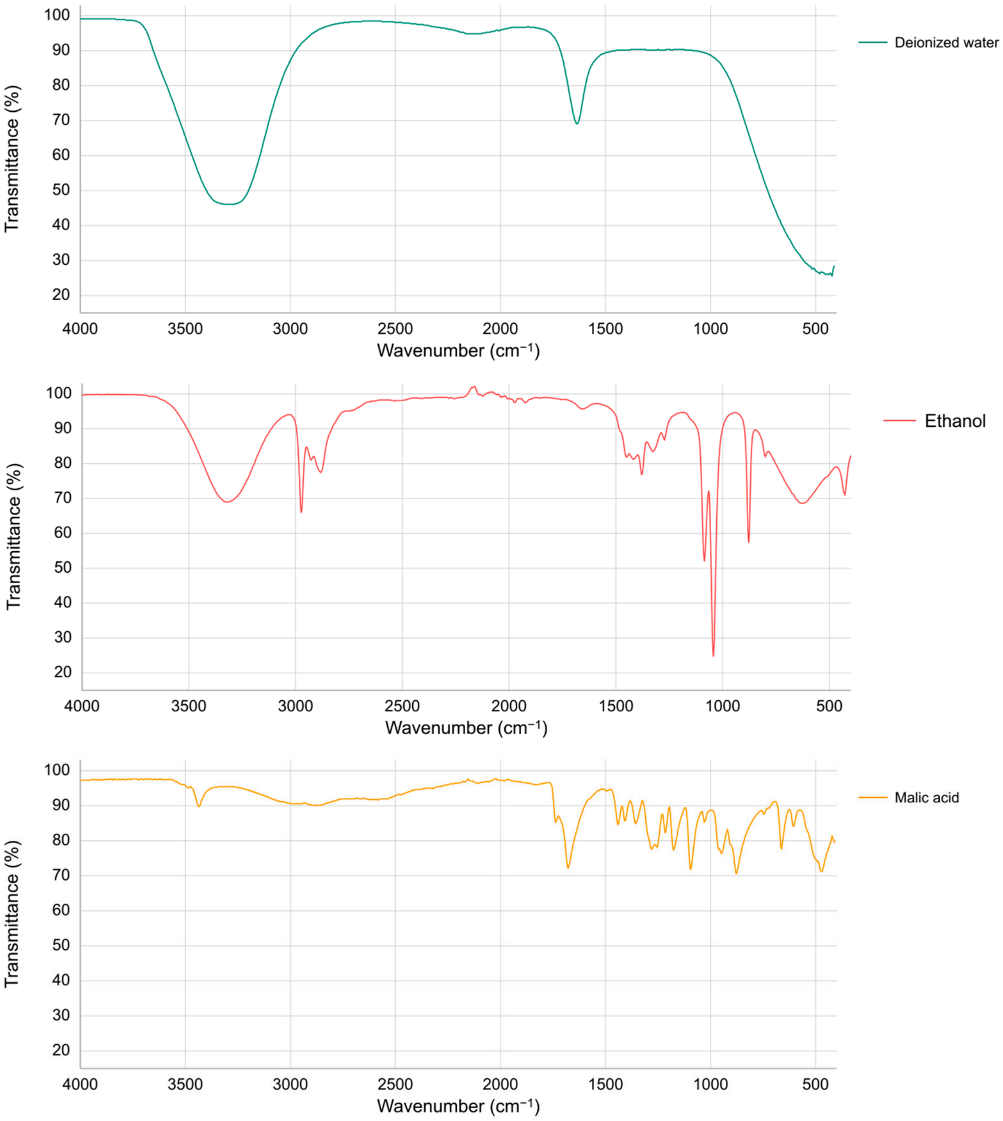

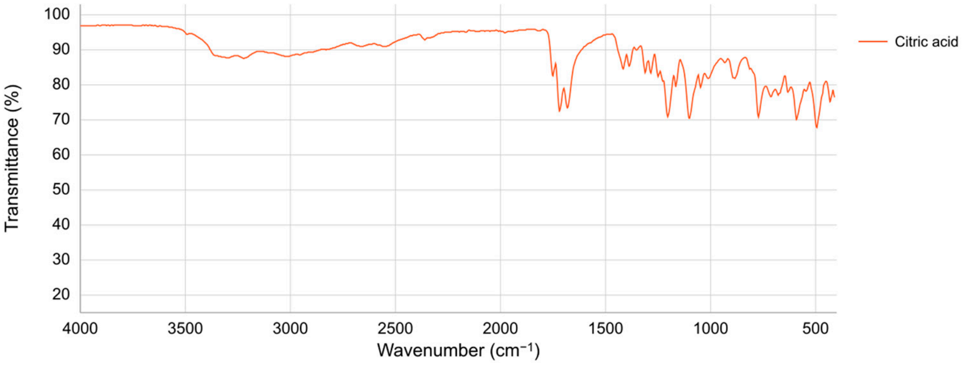

Continuation of Figure S2

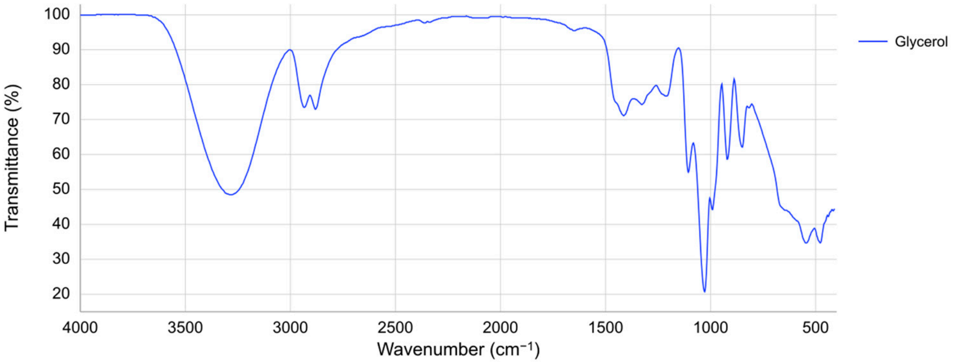

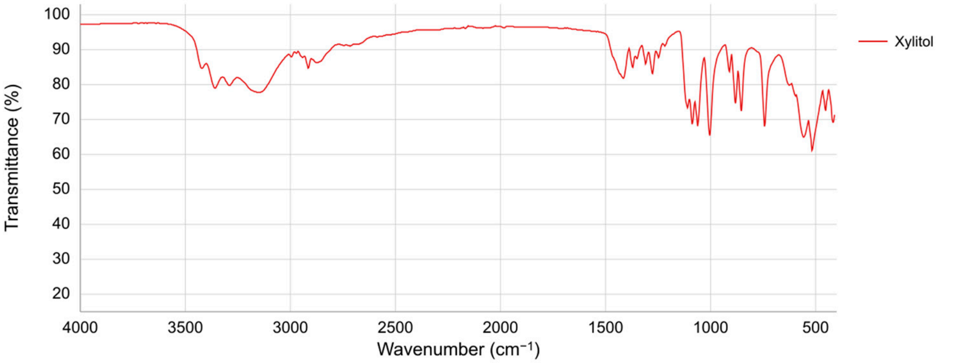

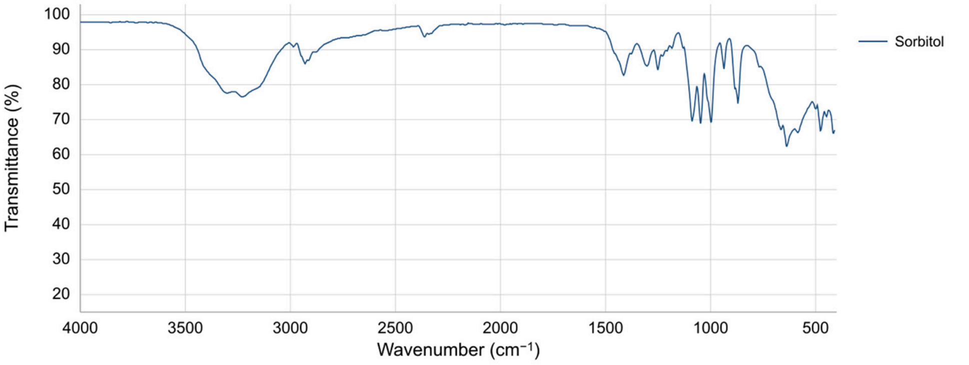

Figure S2. ATR-FTIR spectra of deionized water, absolute ethanol, malic acid, citric acid, glycerol, xylitol, and sorbitol. Deionized water, the organic acids, and the polyols were used to prepare the investigated NADES formulations, whereas absolute ethanol was used as a conventional extraction solvent.

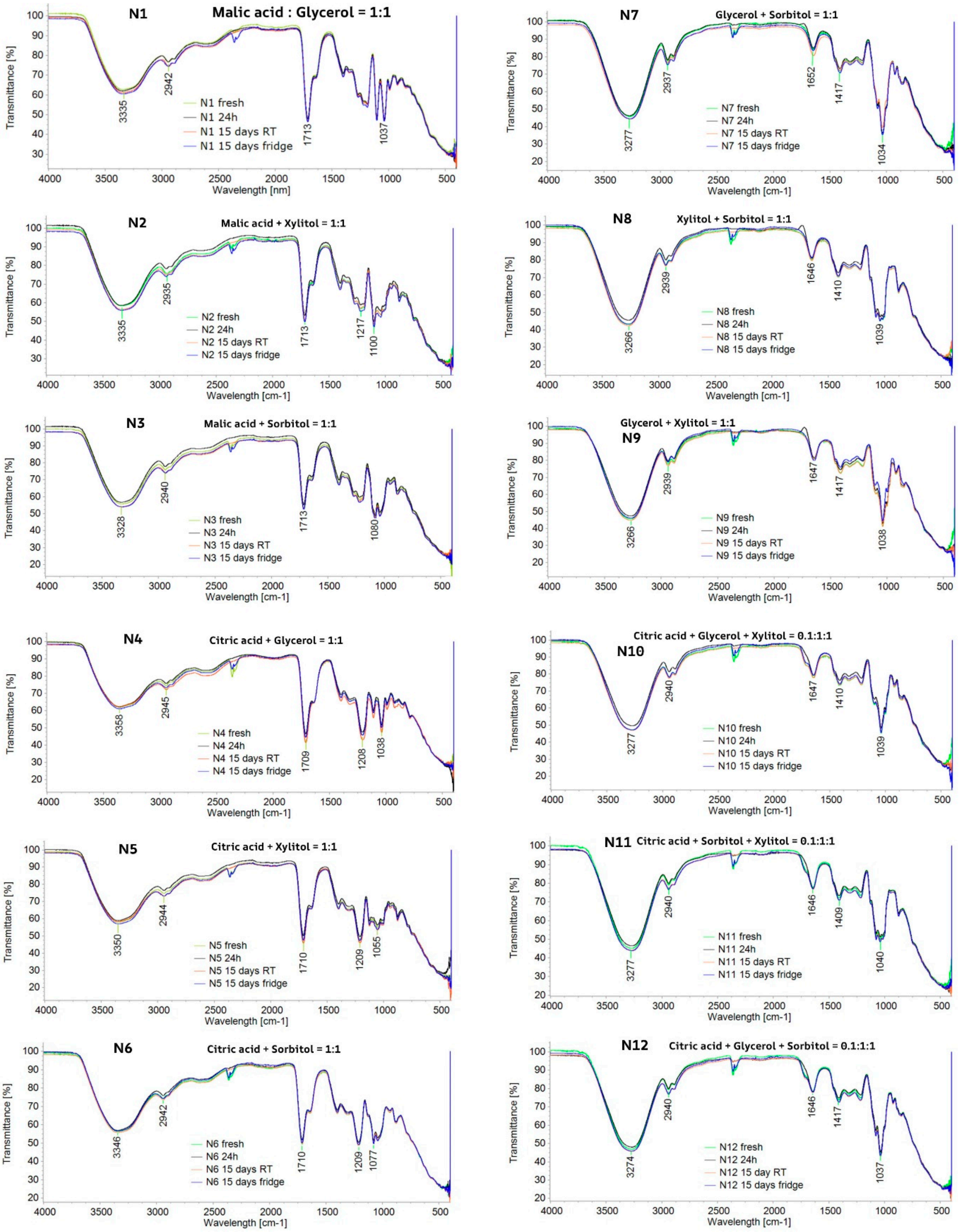

Figure S3. ATR-FTIR spectra of neat NADES formulations N1-N12 recorded as fresh samples, after 24 h, after 15 days at room temperature, and after 15 days under refrigeration.

For each NADES formulation, the spectra recorded immediately after preparation, after 24 h, and after 15 days at room temperature or under refrigeration showed substantial overlap, with only minor intensity variations and no appreciable band shifts or newly appearing spectral features (Figure S3).

Table S1. Apparent pH measurements of NADES, NADES-based and ethanolic extracts of *Nigella sativa*

NADES #	Apparent pH NADES	Extract #	Apparent pH of Extracts
N1	0.18	E1	0.48
N2	0.51	E2	0.65
N3	0.51	E3	0.68
N4	0.12	E4	0.2
N5	0.28	E5	0.35
N6	0.35	E6	0.42
N7	2.38	E7	3.28
N8	3.0	E8	4.21
N9	4.52	E9	4.71
N10	1.78	E10	2.27
N11	1.86	E11	2.27
N12	1.76	E12	2.17
Ultrasound-base EtOH extract		E13	5.0
Soxhlet based EtOH extract		E14	5.8

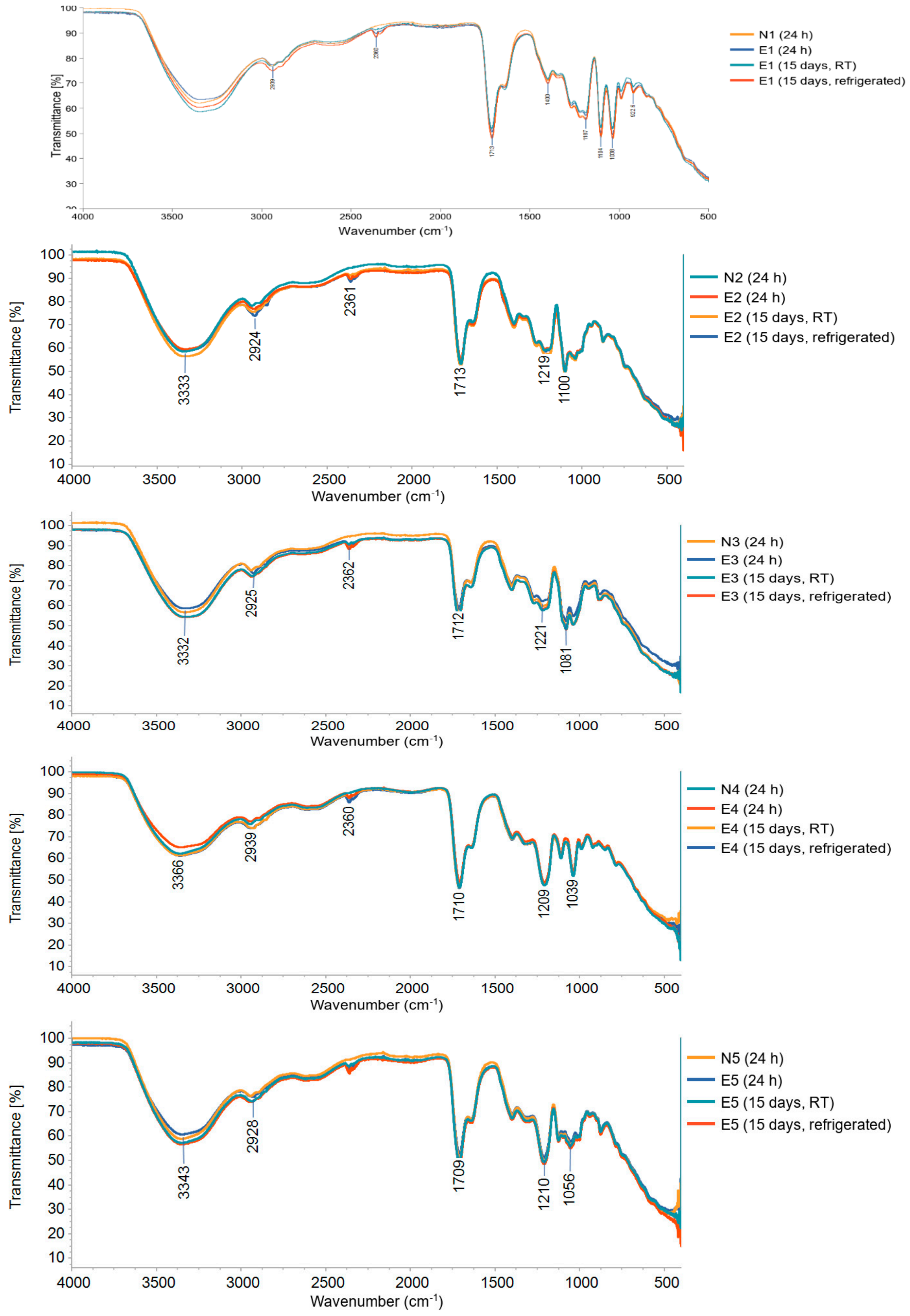
Transmittance [%]
Wavenumber (cm-1)
N1 (24 h)
E1 (24 h)
E1 (15 days, RT)
E1 (15 days, refrigerated)
N2 (24 h)
E2 (24 h)
E2 (15 days, RT)
E2 (15 days, refrigerated)
3333
2924
2361
1713
1219
1100
N3 (24 h)
E3 (24 h)
E3 (15 days, RT)
E3 (15 days, refrigerated)
3332
2925
2362
1712
1221
1081
N4 (24 h)
E4 (24 h)
E4 (15 days, RT)
E4 (15 days, refrigerated)
3366
2939
2360
1710
1209
1039
N5 (24 h)
E5 (24 h)
E5 (15 days, RT)
E5 (15 days, refrigerated)
3343
2928
1709
1210
1056

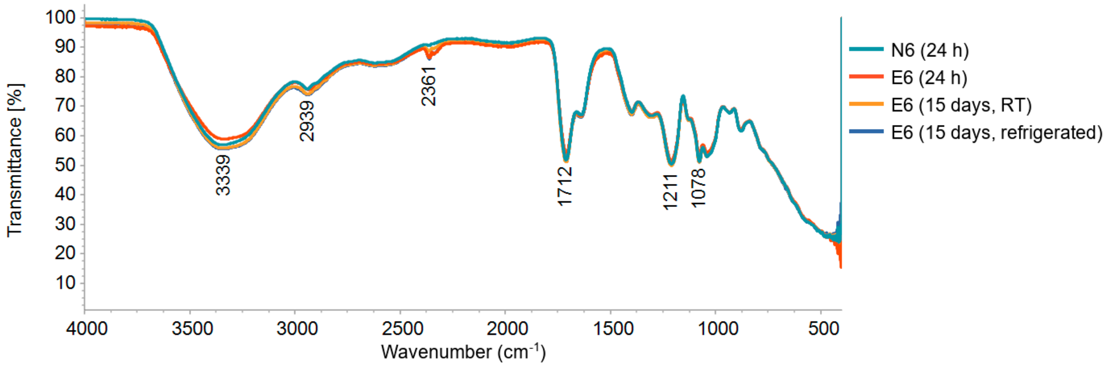
Transmittance [%]
Wavenumber (cm-1)
3339
2939
2361
1712
1211
1078
N6 (24 h)
E6 (24 h)
E6 (15 days, RT)
E6 (15 days, refrigerated)

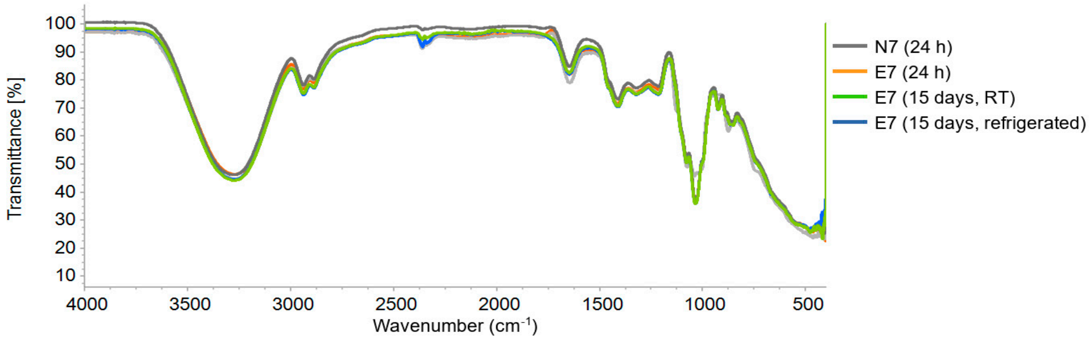
Transmittance [%]
Wavenumber (cm-1)
N7 (24 h)
E7 (24 h)
E7 (15 days, RT)
E7 (15 days, refrigerated)

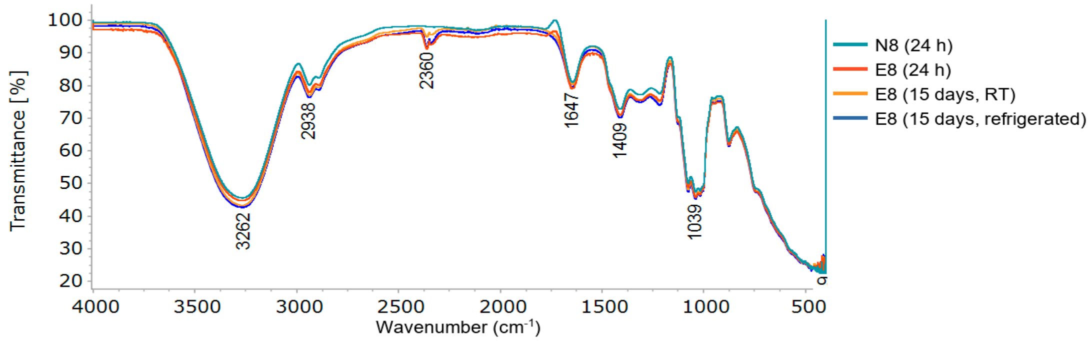
Transmittance [%]
Wavenumber (cm-1)
3262
2938
2360
1647
1409
1039
N8 (24 h)
E8 (24 h)
E8 (15 days, RT)
E8 (15 days, refrigerated)

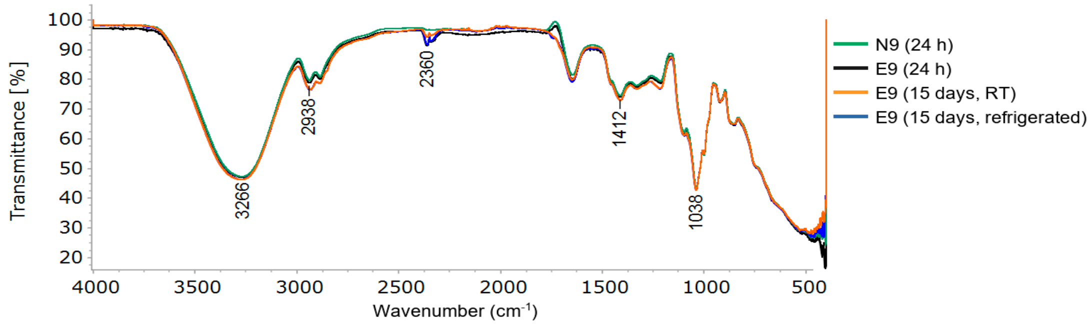
Transmittance [%]
Wavenumber (cm-1)
3266
2938
2360
1412
1038
N9 (24 h)
E9 (24 h)
E9 (15 days, RT)
E9 (15 days, refrigerated)

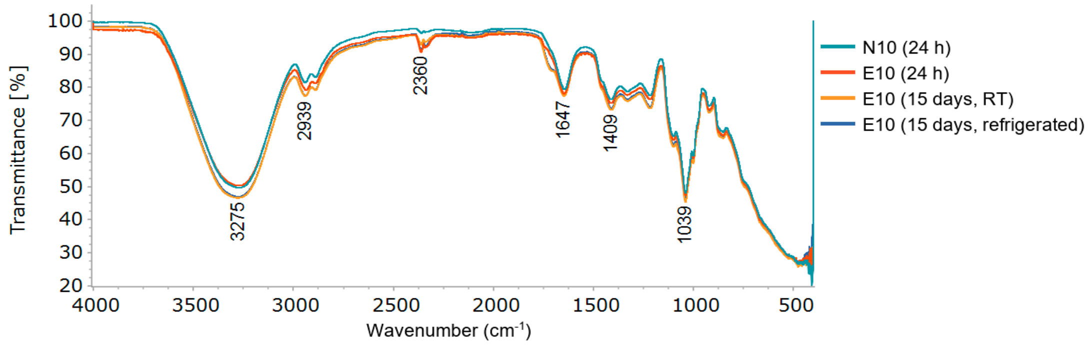
Transmittance [%]
Wavenumber (cm-1)
3275
2939
2360
1647
1409
1039
N10 (24 h)
E10 (24 h)
E10 (15 days, RT)
E10 (15 days, refrigerated)

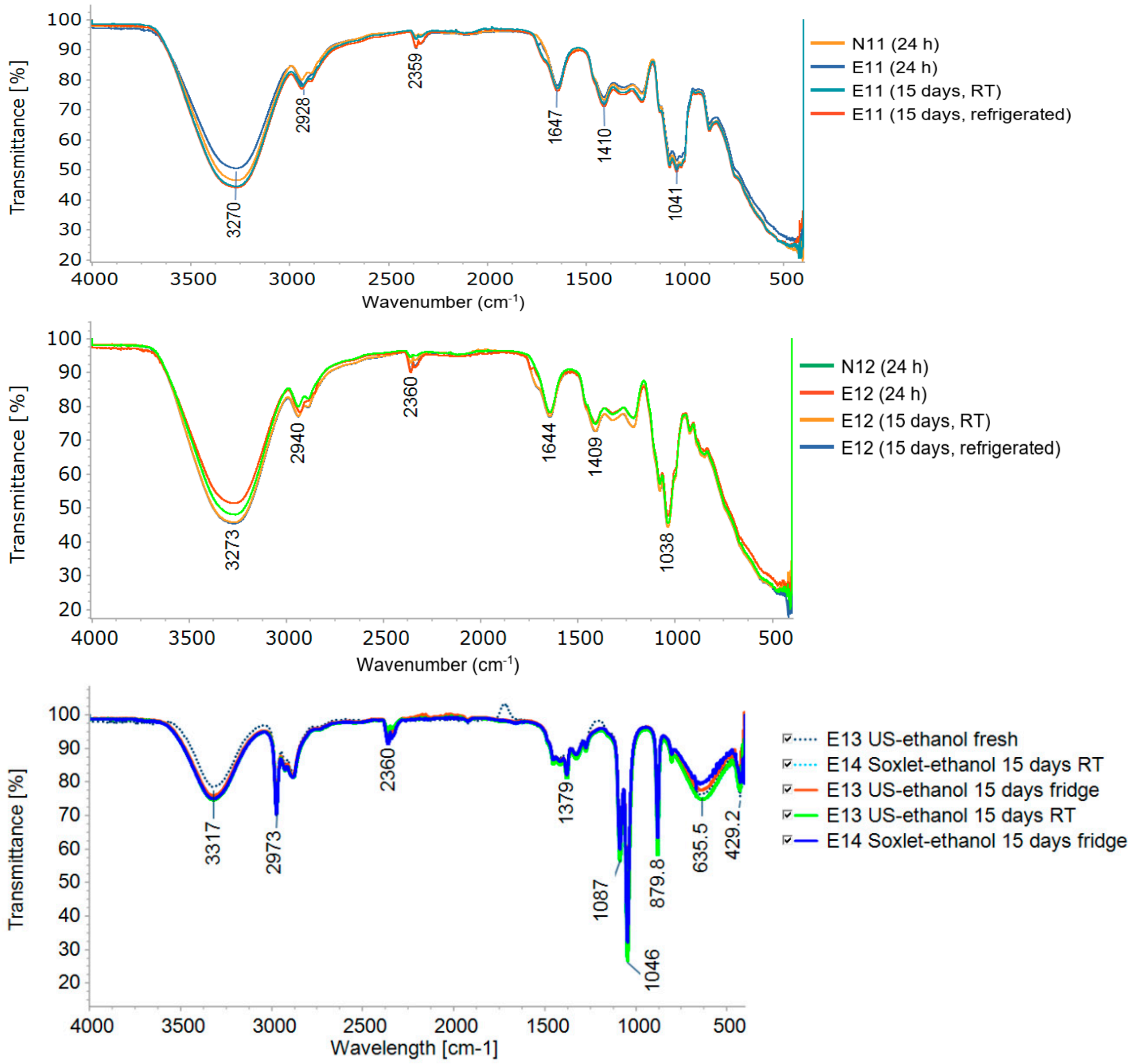

Figure S4. ATR-FTIR spectra of the designed NADES formulations (N1–N12) after 24 h and their corresponding ultrasound-assisted *Nigella sativa* extracts (E1–E12) after 24 h and 15 days of storage at room temperature or under refrigeration. The final panel shows the conventional ethanolic extracts obtained by ultrasound-assisted extraction (E13) and Soxhlet extraction (E14) under the available storage conditions. Only minor variations in relative band intensities were observed, while the principal absorption bands remained unchanged.

2.3. DSC Characterization of NADES and NADES-Based Extracts

Table S2. DSC integration intervals, peak temperatures, and integrated heat values of the neat NADES formulations and their corresponding *Nigella sativa* extracts.

Sample	Event	Integration interval (°C)	T_{peak} (°C)	Integrated heat (J g^{-1})
N1	1	-4.39 to 5.88	1.784	4.396
N1	2	16.48 to 37.08	26.020	8.063
N1	3	112.41 to 229.28	150.221	-59.761
E1	1	58.52 to 109.60	77.637	24.735
E1	2	116.70 to 156.15	128.028	70.120

Sample	Event	Integration interval (°C)	T_{peak} (°C)	Integrated heat (J g⁻¹)
N2	1	-1.25 to 6.38	1.740	1.171
N2	2	20.11 to 31.10	22.795	7.966
N2	3	109.05 to 249.11	127.759	528.404
E2	1	7.48 to 18.65	12.971	5.102
E2	2	81.21 to 131.01	84.085	150.635
E2	3	130.96 to 174.04	141.853	207.031
N3	1	22.27 to 33.49	25.100	1.767
N3	2	109.43 to 154.26	136.350	9.239
N3	3	154.39 to 240.82	189.417	78.550
E3	1	-5.45 to 5.85	1.726	4.199
E3	2	5.85 to 29.60	17.588	12.835
E3	3	42.52 to 53.51	44.548	4.727
E3	4	66.63 to 88.36	76.111	4.630
E3	5	111.52 to 133.33	131.385	17.953
E3	6	161.96 to 223.56	204.068	21.842
N4	1	5.89 to 21.94	11.749	5.315
N4	2	53.96 to 127.27	110.633	38.570
N4	3	129.52 to 258.45	190.614	182.308
E4	1	17.79 to 33.73	22.545	22.972
E4	2	34.29 to 97.63	70.326	183.052
E4	3	101.16 to 150.90	126.544	101.163
N5	1	-2.07 to 6.59	1.902	0.728
N5	2	11.21 to 23.85	17.650	2.056
N5	3	69.13 to 260.20	182.492	366.401
E5	1	2.48 to 11.10	4.720	0.631
E5	2	48.02 to 255.60	182.354	436.341
N6	1	5.60 to 12.12	6.299	1.482
N6	2	73.29 to 243.70	173.714	351.513
E6	1	6.49 to 17.52	9.455	2.003
E6	2	22.80 to 40.34	28.332	5.297
E6	3	66.09 to 140.67	107.389	19.878
E6	4	149.20 to 235.52	182.396	153.293
N7	1	-4.30 to 5.95	-2.009	3.781
N7	2	41.89 to 194.16	118.566	157.716
E7	1	28.52 to 47.07	32.662	3.482
E7	2	51.23 to 62.02	55.675	3.213
E7	3	65.03 to 73.56	68.981	1.851
E7	4	103.08 to 201.56	140.697	202.268
N8	1	5.23 to 16.82	13.957	0.743

Sample	Event	Integration interval (°C)	T_{peak} (°C)	Integrated heat (J g^{-1})
N8	2	19.50 to 39.97	22.136	2.581
N8	3	42.31 to 223.53	134.497	174.361
E8	1	6.24 to 25.47	15.388	7.829
E8	2	26.83 to 222.06	121.633	246.699
N9	1	-0.06 to 6.91	2.612	0.794
N9	2	33.23 to 102.95	95.868	61.094
N9	3	104.13 to 193.77	139.920	33.671
N9	4	193.41 to 255.08	196.408	179.305
E9	1	43.51 to 80.90	56.657	15.221
E9	2	85.20 to 99.33	93.598	6.129
E9	3	107.29 to 218.33	130.772	151.112
E9	4	224.77 to 255.63	246.200	13.452
N10	1	1.27 to 6.08	3.135	1.637
N10	2	37.12 to 193.63	130.081	592.128
E10	1	6.52 to 23.33	14.160	26.717
E10	2	40.66 to 76.45	50.423	11.288
E10	3	94.97 to 196.89	133.464	227.396
N11	1	10.23 to 21.59	11.287	2.277
N11	2	22.17 to 174.96	92.254	557.864
E11	1	-1.04 to 5.58	1.586	2.511
E11	2	37.89 to 189.98	126.801	128.110
E11	3	194.72 to 235.74	210.409	6.012
N12	1	-4.30 to 6.10	1.137	1.223
N12	2	30.45 to 170.09	76.344	453.406
N12	3	171.37 to 269.05	225.434	97.834
E12	1	14.70 to 29.20	19.847	17.727
E12	2	90.04 to 161.14	142.271	441.591
E12	3	169.76 to 235.70	207.282	58.494

Integration intervals correspond to the baseline endpoints annotated in CALISTO and do not represent extrapolated onset and offset temperatures. Because several events were broad, asymmetric, or overlapping, software-generated tangent onset and offset values were not retained. Peak temperatures and integrated heat values were transcribed directly from the original annotated thermograms. For E5, a nested secondary integration of the same broad high-temperature event was omitted to avoid duplicate reporting.

2.4. Physicochemical Properties and Oxidative Quality of Cold-Pressed Oils

To provide a benchmark for comparison with the NADES- and ethanol-derived extracts, five cold-pressed *Nigella sativa* oils, comprising one in-house preparation and four commercially available products, were evaluated with respect to their physicochemical properties, antioxidant capacity, oxidative status, and fatty acid composition. While the oils exhibited relatively similar bulk physical

characteristics, substantial differences were observed in antioxidant capacity and conventional quality indices, indicating considerable variability in overall product quality despite a common botanical origin (Table S3).

Table S3. Physicochemical quality, antioxidant capacity, and oxidative quality indices of the analyzed cold-pressed *Nigella sativa* oils.

Sample	Dynamic viscosity (mPa·s)	Kinematic viscosity (mm^2/s)	FRAP (μmol Fe/100 g oil)	DPPH IC_{50} (mg oil mL^{-1})	Free acidity (% oleic acid)	Peroxide value (meq O_2 kg^{-1})	p-Anisidine value	Iodine value (g I_2/100 g oil)	SAP value (mg KOH g^{-1} oil)
In-house oil	31.7 ± 1.5	34.9 ± 2.5	3894 ± 9	51.2		12.2 ± 0.3		120.6 ± 0.1	175.0 ± 0.6
Oil 2	29.2 ± 1.3	33.0 ± 2.0	9015 ± 3	8.9		10.9 ± 0.1		123.3 ± 1.4	173.6 ± 2.6
Oil 3	28.9 ± 2.9	32.3 ± 4.4	3017± 1	44.0		9.4 ± 0.3		120.9 ± 0.2	174.4 ± 0.3
Oil 4	31.6 ± 1.2	34.4 ± 2.0	3640 ± 3	49.1		29.5 ± 0.8		123.8 ± 1.5	165.5 ± 0.6
Oil 5	26.1 ± 2.2	29.3 ± 3.0	3815 ± 6	15.8		19.7 ± 0.7		124.6 ± 0.2	175.0 ± 2.5

Values shown with the ± symbol are presented as mean ± standard deviation from triplicate measurements. DPPH radical-scavenging activity is expressed as IC_{50} (mg oil mL^{-1}), where lower values indicate higher antioxidant activity; the available DPPH IC_{50} results are reported as single derived values. The p-anisidine value is dimensionless. SAP – saponification.

The dynamic viscosity of the analyzed oils varied only moderately, ranging from 26.1 ± 2.2 to 31.7 ± 1.5 mPa·s, while kinematic viscosity ranged from 29.3 ± 3.0 to 34.9 ± 2.5 mm^2 s^{-1}, indicating broadly similar flow behavior among samples (Table S3). In contrast, antioxidant capacity differed considerably. Ferric reducing antioxidant power (FRAP) ranged from 3017 ± 1 to 9015 ± 3 μmol Fe/100 g oil, with the highest value observed for Oil 2 and the lowest for Oil 3. Likewise, DPPH radical-scavenging activity, expressed as IC_{50}, ranged from 8.9 to 51.2 mg oil mL^{-1}, with Oil 2 exhibiting the lowest IC_{50} value (highest radical-scavenging activity), followed by Oil 5, whereas the In-house oil and Oil 4 displayed the highest IC_{50} values, indicating comparatively lower antioxidant activity. These findings demonstrate that, despite relatively similar physical properties, the antioxidant potential differed substantially among the analyzed oils.

Conventional quality indices likewise showed pronounced variability (Table S3). Free acidity ranged from 2.8 ± 0.0 to 14.5 ± 0.1% (as oleic acid), with the lowest value observed for Oil 4 and the highest for the In-house oil. Peroxide values ranged from 9.4 ± 0.3 to 29.5 ± 0.8 meq O_2 kg^{-1} oil, with the lowest value recorded for Oil 3 and the highest for Oil 4, while p-anisidine values ranged from 0.0 to 48.0, indicating marked differences in the accumulation of secondary oxidation products. In contrast, iodine values (120.6–124.6 g I_2/100 g oil) and saponification values (165.5–175.0 mg KOH g^{-1} oil) varied comparatively little among the analyzed oils (Table S3).

2.5. Fatty Acid Composition of Selected Cold-Pressed Oils

To evaluate the influence of suspended seed particles on oil composition, the In-house oil and Oil 3 were additionally analyzed before and after centrifugation. Only minor changes were observed in refractive index following centrifugation, while gravimetric moisture and Karl Fischer water contents differed primarily because gravimetric drying also accounts for volatile constituents and particle-associated moisture (Table S4).

Table S4. Physicochemical properties of the in-house oil and Oil 3 before and after centrifugation.

Parameter	Unit	In-house oil (non-centrifuged)	In-house oil (centrifuged)	Oil 3 (non-centrifuged)	Oil 3 (centrifuged)
Refractive index (20 °C)	–	1.4723	1.4725	1.4740	1.4740
Moisture (gravimetric)	%	0.48	–	3.22	–
Water content (Karl Fischer)	%	0.14	–	0.10	–

Physicochemical properties of the in-house oil and Oil 3 before and after centrifugation. Moisture content was determined gravimetrically, while water content was measured by Karl Fischer titration. Refractive indices were measured at 20 °C. Data are summarized from the analytical laboratory reports.

GC-FID analysis showed that both oils were dominated by linoleic acid, followed by oleic and palmitic acids, consistent with the characteristic fatty acid profile reported for *Nigella sativa* oil [29] (Table S5). The individual determinations showed numerically similar fatty acid profiles before and after centrifugation, suggesting no apparent change in the relative abundance of the major fatty acids; however, the absence of replicate measurements precludes statistical confirmation of the effects of centrifugation or suspended seed particles on the overall lipid composition.

Table S5. Fatty acid composition of the in-house oil and Oil 3 before and after centrifugation determined by GC-FID (g/100 g total fatty acids).

Fatty acid	In-house oil	In-house oil (centrifuged)	Oil 3	Oil 3 (centrifuged)
C14:0	0.24	0.22	0.21	0.20
C16:0	12.84	12.40	12.02	11.96
C16:1	0.26	0.27	0.28	0.25
C18:0	3.36	3.31	2.77	2.79
C18:1 trans	0.16	0.16	0.15	0.15
C18:1 cis (oleic acid)	25.34	25.68	22.91	23.00
C18:2 cis (linoleic acid)	53.62	53.96	56.80	56.76
C20:0	0.32	0.30	0.28	0.31
C20:1	0.31	0.30	0.38	0.38
C18:3 (ALA)	0.20	0.20	0.25	0.25
C21:0	0.41	0.30	0.50	0.52
C20:2	2.30	2.31	2.77	2.75
C22:2	0.12	0.10	0.13	0.12

Major fatty acid composition of the in-house oil and Oil 3 before and after centrifugation determined by GC-FID. Fatty acids present at concentrations below 0.1 g/100 g total fatty acids are omitted for clarity because they remained below the detection limit in all analyzed samples. Values are expressed as g/100 g total fatty acids, and represent individual compositional determinations obtained from the analytical laboratory reports; therefore, standard deviations and inferential statistical comparisons are not provided.

molecules

MDPI

2.6. Phenolic Content, Antioxidant Activity, and Cytocompatibility of the Investigated Extracts

Table S6. Total phenolic content (TPC) and ABTS antioxidant activity of the investigated ethanolic extracts, NADES-based extracts, and cold-pressed oils, with Šídák-adjusted pairwise comparisons against Soxhlet ethanolic extraction.

Sample	TPC (mg GAE/g seed), mean ± SD	TPC vs Soxhlet-EtOH, adjusted p	ABTS (µmol TE/g seed), mean ± SD	ABTS vs Soxhlet-EtOH, adjusted p
UAE-EtOH	1.275 ± 0.107	Lower; <0.0001 (****)	10.77 ± 0.22	Lower; <0.0001 (****)
Soxhlet-EtOH	1.738 ± 0.152	Reference	32.75 ± 0.58	Reference
E1	2.153 ± 0.114	Higher; <0.0001 (****)	50.25 ± 1.07	Higher; <0.0001 (****)
E2	1.543 ± 0.092	ns; 0.8149	22.55 ± 0.60	Lower; <0.0001 (****)
E3	1.353 ± 0.106	Lower; 0.0004 (***)	29.10 ± 1.40	ns; 0.3106
E4	1.715 ± 0.102	ns; >0.9999	23.93 ± 2.30	Lower; <0.0001 (****)
E5	1.408 ± 0.060	Lower; 0.0054 (**)	34.58 ± 1.14	ns; >0.9999
E6	1.225 ± 0.181	Lower; <0.0001 (****)	26.73 ± 0.64	Lower; 0.0003 (***)
E7	0.813 ± 0.099	Lower; <0.0001 (****)	29.48 ± 2.50	ns; 0.6153
E8	0.640 ± 0.033	Lower; <0.0001 (****)	22.73 ± 0.82	Lower; <0.0001 (****)
E9	1.335 ± 0.132	Lower; 0.0002 (***)	64.28 ± 1.94	Higher; <0.0001 (****)
E10	1.588 ± 0.130	ns; 0.9996	40.83 ± 3.13	Higher; <0.0001 (****)
E11	1.188 ± 0.053	Lower; <0.0001 (****)	33.95 ± 3.89	ns; >0.9999
E12	1.710 ± 0.196	ns; >0.9999	43.48 ± 1.59	Higher; <0.0001 (****)
Oil 1	0.063 ± 0.010	Lower; <0.0001 (****)	1.15 ± 0.13	Lower; <0.0001 (****)
Oil 2	0.083 ± 0.029	Lower; <0.0001 (****)	3.20 ± 0.18	Lower; <0.0001 (****)
Oil 3	0.125 ± 0.013	Lower; <0.0001 (****)	1.02 ± 0.15	Lower; <0.0001 (****)
Oil 4	0.473 ± 0.015	Lower; <0.0001 (****)	2.45 ± 0.06	Lower; <0.0001 (****)
Oil 5	0.158 ± 0.015	Lower; <0.0001 (****)	1.25 ± 0.10	Lower; <0.0001 (****)

Sidak-adjusted p-values following ordinary one-way ANOVA; n = 4. Higher/Lower indicates the direction relative to Soxhlet-EtOH. ns, not significant; * $p < 0.05$; ** $p < 0.01$; *** $p < 0.001$; **** $p < 0.0001$.

2.7. Effect of Partial Neutralization on Cell Viability

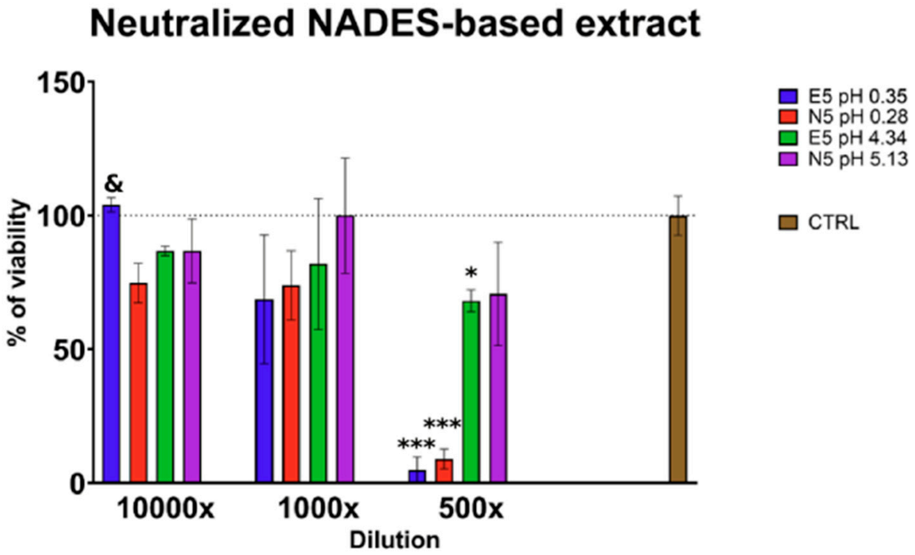

Figure S5. Effect of partial pH neutralization on the viability of HaCaT cells exposed to the selected *Nigella sativa* NADES formulation and its corresponding extract. NADES N5 (initial pH 0.28) and its corresponding extract E5 (initial pH 0.35) were partially neutralized with concentrated NaOH to pH 5.13 and 4.34, respectively. Cell viability was determined after exposure to 10,000×, 1,000×, and 500× dilutions. Partial neutralization markedly improved cell viability at the 500× dilution, whereas only minor differences were observed at 10,000× and 1,000×. The dashed line indicates the untreated control (100% viability). Statistical significance relative to the untreated control is indicated as * ($p < 0.05$), *** (p and < 0.001) (Šídák's multiple-comparisons test). The symbol **&** denotes a statistically significant difference between acidic E5 and its corresponding neat NADES (N5) at the same dilution.
